# Multiple claudin–claudin *cis* interfaces are required for tight junction strand formation and inherent flexibility

**DOI:** 10.1038/s42003-018-0051-5

**Published:** 2018-05-17

**Authors:** Jun Zhao, Evan S. Krystofiak, Angela Ballesteros, Runjia Cui, Christina M. Van Itallie, James M. Anderson, Cristina Fenollar-Ferrer, Bechara Kachar

**Affiliations:** 1 Laboratory of Cell Structure and Dynamics, National Institute on Deafness and Other Communication Disorders, 35A Convent Drive, Bethesda, MD 20892 USA; 2 Laboratory of Tight Junction Structure and Function, National Heart, Lung, and Blood Institute, 50 South Drive, Bethesda, MD 20892 USA; 3 Computational Structural Biology Unit, National Institute of Neurological Disorders and Stroke, 35 Convent Drive, Bethesda, MD 20892 USA; 4 Laboratory of Molecular & Cellular Neurobiology, National Institute of Mental Health, 35 Convent Drive, Bethesda, MD 20892 USA; 5 Present Address: Cancer and Inflammation Program, National Cancer Institute, Frederick, MD 21702 USA; 6 Present Address: Molecular Physiology and Biophysics Section, National Institute of Neurological Disorders and Stroke, Bethesda, MD 20892 USA

## Abstract

Tight junctions consist of a network of sealing strands that create selective ion permeability barriers between adjoining epithelial or endothelial cells. The current model for tight junction strands consists of paired rows of claudins (Cldn) coupled by a *cis* interface (X-1) derived from crystalline Cldn15. Here we show that tight junction strands exhibit a broad range of lateral bending, indicating diversity in *cis* interactions. By combining protein–protein docking, coevolutionary analysis, molecular dynamics, and a mutagenesis screen, we identify a new Cldn–Cldn *cis* interface (Cis-1) that shares interacting residues with X-1 but has an ~ 17° lateral rotation between monomers. In addition, we found that a missense mutation in a Cldn14 that causes deafness and contributes stronger to Cis-1 than to X-1 prevents strand formation in cultured cells. Our results suggest that Cis-1 contributes to the inherent structural flexibility of tight junction strands and is required for maintaining permeability barrier function and hearing.

## Abstract (web summary)

Jun Zhao, Evan S. Krystofiak, and colleagues identified a new cis interface (Cis-1) essential for the formation of normal tight junctions. This study suggests that Cis-1 contributes to maintaining structural flexibility of tight junction strands for proper ion balance and hearing.

## Introduction

Tight junctions form intercellular barriers that regulate paracellular ion permeation in a diverse array of epithelial and endothelial tissues. Tight junctions consist of networks of linear sealing strands between adjoining cells, typically composed of claudins^1^ (Cldn) and several other transmembrane (TM) proteins including occludin^2^ and tricellulin^3^. Cldns are considered the principal structural components of tight junctions, because they are able to form strands in the absence of other tight junction proteins when expressed in heterologous systems^4^. All members of the Cldn family have four TM domains, intracellular N- and C-terminal ends, and two extracellular loops (ECL)^5^. The first ECL (ECL1) is longer and contains five β-strands forming an antiparallel β-sheet and an extracellular helix (ECH), whereas the shorter second ECL (ECL2) exhibits a helix-turn-helix motif^6, 7^. Residues on both ECLs have been implicated in Cldn strand formation^7–11^. For tight junction strand formation, each Cldn monomer must not only interact with neighboring Cldn within the same cell membrane (*cis*) but also with Cldns across the extracellular space on a neighboring cell membrane (*trans*).

The recently reported crystal structures of the mouse claudin-15 (mCldn15) monomer (PDB ID:4P79)^6^ and the claudin-19 and claudin-4 complexes with the *Clostridium perfringens* enterotoxin (CPE) (PDB ID:3 × 29 and 5B2G)^12, 13^ have provided structural information to model tight junction strands. Suzuki et al.^9^ proposed a tight junction strand model featuring an antiparallel double row of Cldn in opposing plasma membranes. The model contains a medial-cis interaction between the ECL1 β-sheets that was further substantiated by mutagenesis of key residues in this region^11^. This model depends on an axial cis interface (X-1) that involves a hydrophobic interaction between the methionine 68 (M68) on the ECH and two phenylalanines (F146 and F147) on ECL2^6, 9^. This double-rowed model is consistent with biochemical analysis of Cldn strands^8, 14^ and shows a possible ion permeability mechanism. However, the X-1 interface was generated using the crystal structure and based mainly on the crystal packing observed on the mCldn15 crystal structure without considering the effect of lipid bilayers, the position of residues not resolved in the crystal structure, and the biological flexibility of the Cldn strands.

In this study, we examine the dynamics of Cldn strands by performing live imaging on cells expressing fluorophore-tagged Cldns and measuring their lateral arching and bending within the plane of the membrane. The broad range of curvatures we observe suggests high lateral flexibility or structural variability in the *cis* axial association between Cldn monomers. In addition, we evaluate the proposed Suzuki model^9^ and search for additional cis axial interfaces using computational methods including the following: molecular dynamics, protein–protein docking, and coevolutionary coupled mutations. Key residues for the candidate interfaces were experimentally tested for normal strand formation by point-mutagenesis screening. We identify and validate a new *cis* interface (Cis-1) that presents an ~ 17° rotation in relation to X-1 and is essential for normal strand formation. We show that a deafness-causing missense mutation in human claudin-14 (hCldn14) involves a Cis-1 interface key residue, further validating its biological relevance. This study suggests that X-1 and Cis-1 contribute to tight junction strand structural variability and inherent flexibility.

## Results

### Tight junction strand lateral flexibility

**Fig. 1:**
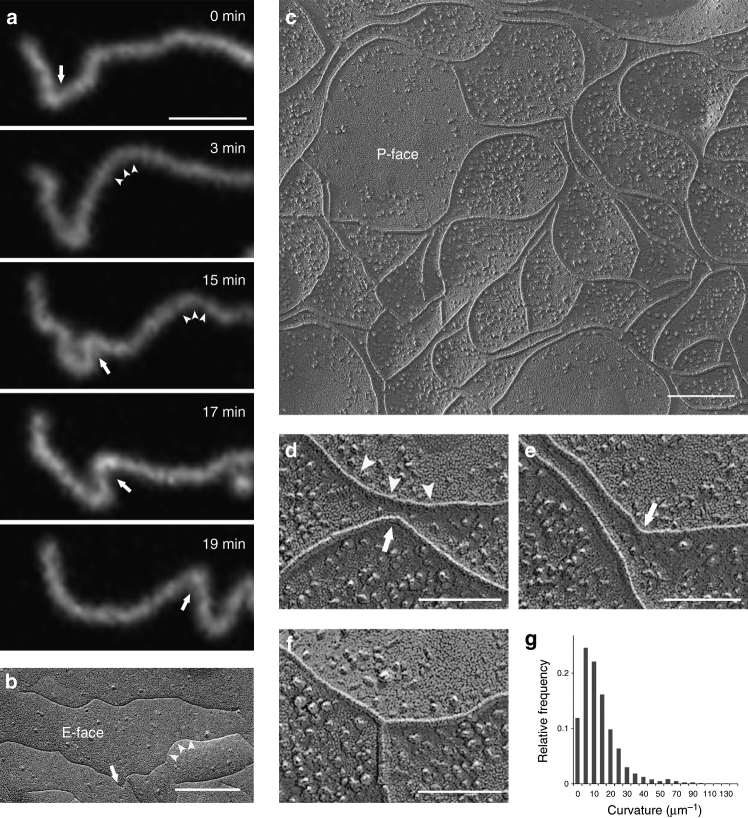
Lateral flexibility of tight junction strands. **a** Fluorescence confocal live imaging of tight junction strands formed between adjacent Rat1 cells expressing mCldn2-GFP. Sequential images show a putative single strand undergoing continuous lateral mobility over the course of minutes. Strands arching and sharp bends in arbitrary directions are indicated with arrowheads and arrows, respectively. Scale bar = 1 µm. **b**–**f** Freeze-fracture TEM of mCldn15 strands expressed in HEK293T cells showing the grooves from single strands on the E-face **b** and a larger interconnected tight junction strand network on the P-face **c**–**f**. **d**, **e** Representative views of strand arching (arrowheads) and discrete bending points (arrows). **f** Example of a branching point. Scale bars = 200 nm for **b**, **c** and 100 nm for **d**–**f**. **g** Histogram showing the distribution of mCld15 strand curvature quantified from freeze-fracture TEM as a function of the inverse radius of curvature, *κ*; *n* = 16 strands

Tight junction assembly^15^ and homeostasis within cellular organizations involves continuous dynamic rearrangements including bending, branching, and possibly annealing of their constituent tight junction intramembrane strands^16, 17^. We analyzed the flexibility of the Cldn strands using live-imaging confocal microscopy of Rat1 cultured cells stably transfected with green fluorescent protein (GFP)-tagged mCldn-2. Rat1 cells are not expected to naturally express tight junction proteins and the heterologous tight junction strands formed are presumed free of other tight junction proteins, similar to other heterologous systems^8, 18^. Rat1 cells form extensive cell–cell lamellipodial contacts where tight junctions form and can be conveniently imaged within the optical plane of view in the confocal microscope^17^. The highly dynamic and flexible nature of tight junction strands can be directly observed in the simplest situation, where the diffraction-limited fluorescence signal of a single strand can be observed isolated from the influence of other neighboring strands (Fig. 1a). Single strands can be observed undergoing changes between straight and curved shapes over the course of minutes, arching and bending laterally in arbitrary directions (Fig. 1a). The arching, inflections, and bending motions cover a broad range of curvatures including sharp kinks (Fig. 1a) and these bending motions appear to progress at different rates (Supplementary Movie 1), suggesting spatiotemporal variabilities in the strand structure and potentially elastic and inelastic remodeling processes.

We quantified the tight junction strand curvature distribution in freeze fracture replicas of HEK293T cells transiently transfected with mCldn15. We examined both loosely packed strands as well as larger strand networks (Fig. 1b, c). Similar to the mCldn-2 strands in live imaging, mCldn15 strands exhibited arches and bends (Fig. 1d, e) in arbitrary directions as well as strands that formed branches (Fig. 1f). Branching and bending were more apparent when strands are clustered at high density (Supplementary Fig. 1a). To derive the distribution of permissible angles between individual mCldn15 protomers in the strand, we measured and plotted the distribution of the radius of curvature along tight junction strands in freeze-fracture replicas (Fig. 1g). The distribution of curvatures for randomly selected tight junction strands ranging in lengths from 0.5 to 1.3 µm (*n* = 16) was determined using the “arcfit” method^19^, to calculate the curvature parameter *κ*, defined as the reciprocal radius of a curvature of a fitted tangential circle^20^. In this representative sample of strands, the curvature distribution ranged from 0 (straight strands) to 123 μm^−1^ (strands that form right-angle bends). The distribution was skewed toward the lower values (straight line) with a median *κ* of 8 μm^−1^ showing that tight junction strands formed by mCldn15 in HEK293T cells are for the most part straight to slightly curved with a minority population of highly curved regions. These curvature values were used to estimate putative angles between protomers based on the inter-monomer distance derived from the proposed crystal structure-based model of the tight junction strand^6^. The distribution showed a median angle of 1.3° per monomer step with 15% of the strand having > 3° per monomer step and extremes of 20° per monomer step. Some of these sharp angles occurred at discrete bending points but others occurred at branching points (Fig. 1d-f). It was difficult to measure curvature at the discrete bending points and these values may be underestimated. We also quantified the distribution of angles at branch points (*n* = 107 branches). For these measurements, we excluded strands that showed a discontinuity at the point of contact, suggesting that it may be an intersection of two independent strands rather than a branch point, and at regions where a single strand appears to symmetrically bifurcate into two strands, described as forking. Branching angles appear as a bimodal population, centered at ~ 67° and 90° (Supplementary Fig. 1). Collectively, these data suggest that the *cis* interaction between Cldn monomers should be either highly compliant to support the diverse curvatures and branching angles or there are multiple forms of *cis* interfaces that support differing angles between Cldn protomers.

### The energetic stability of the double row model

**Fig. 2:**
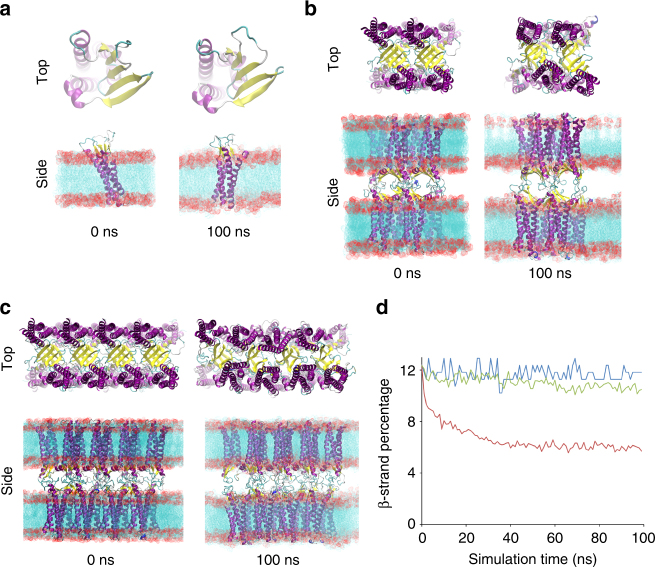
Molecular dynamics simulations show instability in the double row model. The energetic stability of double row mCldn15 model utilizing the X-1 interface was evaluated using molecular dynamics simulations. Top and side views of the initial and final configurations of the monomer **a**, 8 protomer strand **b**, and 16-protomer strand throughout the simulation **c** are shown. mCldn15 protomers are represented as cartoons with the α-helices colored in purple, β-strands in yellow, loops in light blue, and 3–10 helices in dark blue. Lipid bilayers are shown in the side view representation. **d** Time-course plot of the β-structure percentage for the monomer (blue), the 8-protomer strand (green), and 16-protomer strand (red) over the 100 ns molecular dynamics simulations

We evaluated the energetic stability of the mCldn15 crystal structure-based tight junction strand model of Suzuki et al.^9^ in molecular dynamics simulations. We first modeled residues V34 to T41 located in the ECL1 (Supplementary Fig. 2) using a specific loop modeling protocol (see Methods), because they were absent in the mCldn15 crystal structure. The monomeric structure with modeled ECL1 residues was used to build two oligomeric Cldn strands (consisting of 8 protomers and 16 protomers) in double phosphatidylcholine (POPC) lipid bilayers, using the strand model proposed by Suzuki et al.^9^ as a template (Fig. 2 and Supplementary Fig. 2). The monomer and the 8-protomer oligomer did not undergo any large-scale structural re-organization during the molecular dynamics simulations (Fig. 2a, b) in agreement with a recent report that showed that an 8-protomer strand was structurally stable during a similar molecular dynamics simulation^21^. However, the 16-protomer strand underwent rapid re-organization that involved rotations of individual protomers within the membrane plane (Fig. 2c) with an average protomer rotation of 11.3 ± 4.8° at 100 ns relative to their initial positions. For all three molecular dynamics simulations, the β-structure percentage was quantified by measuring the average β-structure percentage of the entire strand throughout the simulation to examine protein unfolding. The monomer and 8-protomer strand had stable β-structures that did not change during the simulation, whereas the 16-protomer strand showed a 40% decrease in the β-sheet configuration compared with its initial value (Fig. 2d). The relative stability of the 8-protomer strand and the loss of structure in the 16-protomer strand show that the strand appears to become increasingly unstable as more protomers are added. Many factors may contribute to the instability of the strand model including the incompatibility with a membrane environment, overtight packing resulting from the additional ECL1 residues, potentially incorrect *cis* or *trans* interfaces, or requirement for flexibility and/or diversity of interfaces.

### Reassessment of residues implicated in *cis* interactions

**Fig. 3:**
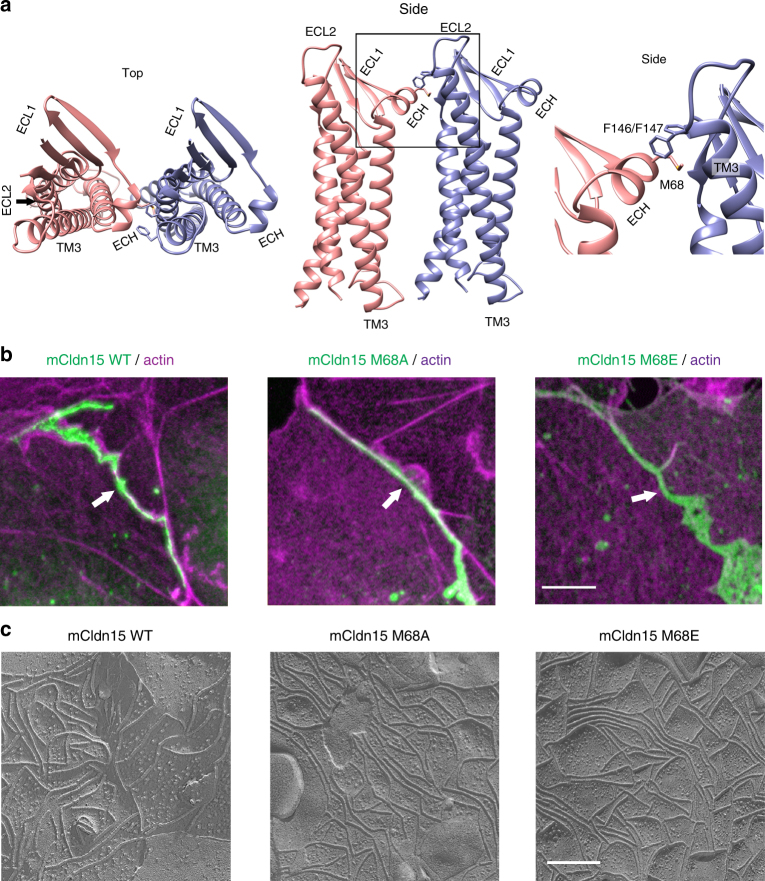
Mutations of residue M68 do not disrupt tight junction strand formation. **a** Top and side view of the X-1 dimer of mCldn15 in cartoon representation, and close view of the indicated box highlighting the previously described ECH/ECL1 and TM3/ECL2 interface region; the side chains of residues M68, F146, and F147 are shown as sticks. **b** Fluorescence confocal images of COS7 cells expressing mCldn15-GFP WT, M68A, and M68E (green) forming tight junctions (arrows) at sites of cell–cell contact. Cellular actin was counterstained with Alexa-405 phalloidin (shown in magenta). **c** Freeze-fracture TEM of HEK293T expressing mCldn15-GFP WT, M68A, and M68E showing the characteristic tight junction strand morphology. Scale bars = 5 µm for confocal images and 200 nm for freeze-fracture TEM images

The instability of the Cldn strand in the molecular dynamics simulation was unexpected, because some features of the X-1 interface have been experimentally validated previously^6^. Consequently, we reexamined the proposed *cis* interface to investigate its biological relevance. Residues, M68, F146, and F147 of mCldn15 were reported to be involved in a hydrophobic interacting network in the X-1 interface that is essential for tight junction strand formation^6^ (Fig. 3a). The original report^6^ used Sf9 insect cells for heterologous mCldn15 expression and screened for tight junction strand formation by freeze fracture. Here we reproduced the same mutations and screened for tight junction formation using COS7 and HEK293T mammalian cells transfected with GFP-tagged mCldn15. Using a combination of fluorescence microscopy and freeze fracture, we confirmed that the F146A single mutant as well as the F146A/F147A double mutant abolished tight junction strand formation (Supplementary Fig. 3). However, we observed that the mCldn15 point mutants M68A and M68E did not prevent formation of tight junction strands and had similar morphology to wild-type (WT) mCldn15 strands. Fluorescence imaging (Fig. 3b) showed comparable frequency of tight junction formation between pairs of adjacent cells expressing the WT (92%), M68A (90%), or M68E (91%) mutants (Supplementary Fig. 4a). Freeze fracture showed the characteristic network of meandering and branching strands (Fig. 3c). This demonstrates that M68 is not essential for mCldn15 strand formation, and that other residues may be involved in *cis*-oligomerization.

### Cldn *cis* interfaces identified using protein–protein docking

**Fig. 4:**
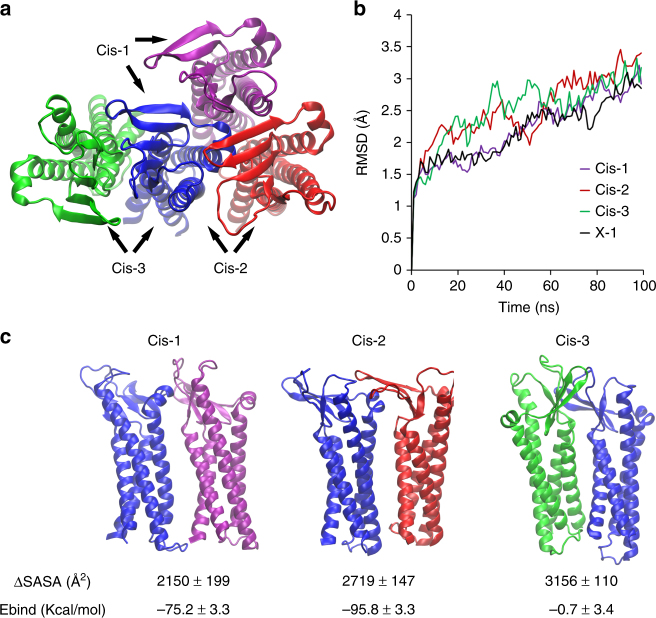
Three candidate *cis* interfaces obtained by in silico mCldn15 protein–protein docking modeling. **a** Top views of the three main mCldn15 dimer poses obtained by “blind” protein–protein docking are shown in cartoon representation and labeled as Cis-1 (blue and purple), Cis-2 (blue and red), and Cis-3 (green and blue). The different interfaces were structurally superimposed using the blue monomer as a reference point. **b** Time-course plot of the RMSD values for Cis-1 (purple), Cis-2 (red), Cis-3 (green), and X-1 (black) dimers over 100 ns molecular dynamics simulations. **c** Side views for Cis-1, Cis-2, and Cis-3 interfaces with their corresponding calculated interface area (∆SASA) and binding energy (Ebind)

We searched for alternative Cldn–Cldn *cis* interfaces using the protein–protein docking algorithm implemented in HADDOCK^22^ without imposing any restraints (blind docking). Out of the initial 1000 runs, 3 clusters that exhibit *cis*-packing were identified. Each cluster, named Cis-1, Cis-2, and Cis-3, is represented by the structure with the lowest energy (Fig. 4a). The Cis-1 interface had a similar overall three-dimensional configuration to that of X-l, but with an ~ 17° rotation between monomers normal to the membrane plane. In Cis-2, the β-strands and TM1 in one monomer interact with the ECH and TM3 on the adjacent monomer (Fig. 4a). In the Cis-3 interaction, the two monomers are oriented as a mirror image of each other, where TM1, TM4, and ECL1 interact with their equivalent in the adjacent monomer (Fig. 4).

In order to further evaluate these three candidate interfaces, we performed 100 ns molecular dynamics simulations for mCld15 dimers in the Cis-1, Cis-2, and Cis-3, as well as the X-1 configuration in a membrane environment generating an ensemble of 5000 structures for each configuration. All four pairs showed root mean square deviation (RMSD) values lower than 5 Å after 30 ns, indicating that they are stable structures in the membrane environment (Fig. 4b). The ensemble of structures from each of the candidate interactions was used to estimate the binding energy (Ebind) and the interface area (∆SASA), measured by the change in solvent accessible surface area (SASA) between two monomers and the dimer configurations. The analysis showed that Cis-1, Cis-2, and Cis-3 have greater ∆SASA (Fig. 4c) than X-1 (331.4 Å^2^). Cis-1 and Cis-2 have more favorable binding energies than X-1 (− 27.3 ± 3.7 kcal/mol), whereas Cis-3 showed unfavorable binding energy (Fig. 4c).

### Coevolutionary coupled mutation analysis

To evaluate the biological likelihood of the three *cis* configurations identified by protein–protein docking, we performed coevolutionary mutation analyses of mCldn15 using the EVfold algorithm^23^. This algorithm identifies  evolutionary coupled pairs of residues (EC pairs) that are usually associated with  key functional or structural positions in proteins, and are often found in close proximity. The EC pairs that are too distant in a monomer can be in close proximity within dimer configurations; constituting dimerization signals can be used to identify intermolecular contacts between a variety of protein oligomers^23–27^.

**Fig. 5:**
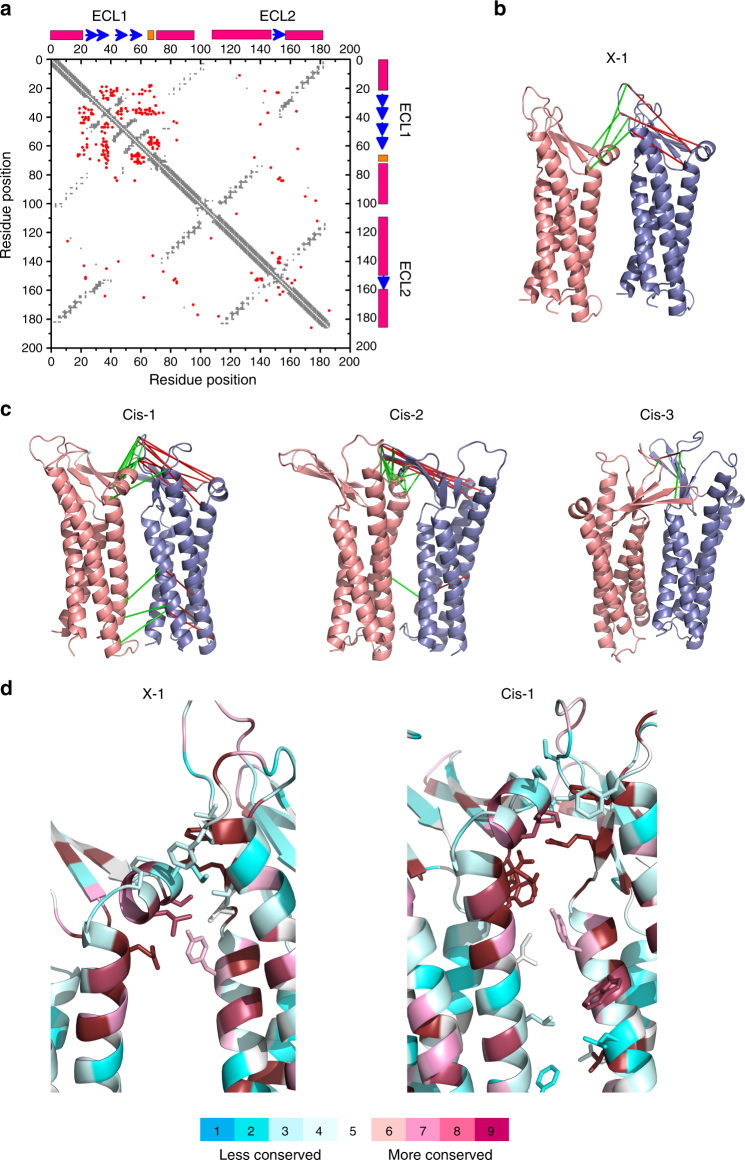
*Cis* interface screening by evolutionary coupling and conservation analysis. **a** Intramolecular contact map obtained from the crystal structure of mCldn15 (PDB ID: 4P79) (gray) and the overlaid intermolecular contact map of mCldn15 identified by coevolutionary coupling analysis (red). The approximate locations of ECL1 and ECL2 and secondary structural elements are indicated along the axis, with TM domains in magenta, β-sheets in blue, and the ECH in orange. Predicted intermolecular residue-residue contacts were mapped onto the X-1 **b** and the three candidate interfaces Cis-1, Cis-2, and Cis-3 **c** obtained from HADDOCK. Intermolecular distance between the EC pairs are shown in green while intra-molecular distance between the same pair are shown in red. **d** Residue conservation of the X-1 and Cis-1 interface, in the region between ECH/ECL1 and ECL2. Side chains of residues within 3 Å of the two Cldn protomers are shown as sticks and color coded by their conservation ratio

In the case of mCldn15, we identified 109 coevolutionary residue pairs with high coupling strengths that did not overlap with the contact map obtained from the Cldn monomer, indicating a likely role in oligomerization (Fig. 5a). The candidate intermolecular EC pairs were mapped onto the three dimer configurations obtained from the blind docking (Cis-1, Cis-2, and Cis-3) as well as X-1 (Fig. 5b, c); only the pairs located directly at each interface were considered. In Cis-1, Cis-2, and the X-1 interfaces, we observed many EC pairs that have much shorter intermolecular distances (< 10 Å) within a dimer than in the corresponding monomeric context, suggesting these residue pairs are likely involved in the dimerization interface. The majority of the EC pairs are located between ECH, ECL1, and ECL2 regions that have been previously reported as important for Cldn oligomerization^7–11^. Cis-3 had only one correlated EC pair and was not further considered as a primary cis interface in the context of this study.

### The Cis-1 interface is conserved in the Cldn family

Residue conservation analysis between members of a protein family can potentially reveal residues that are critical for protein structure and function. In proteins with conserved function, residues involved in substrate binding or protein-protein interactions, not only must be highly conserved, but also accessible to the solvent or membrane environment. Conservation scores obtained using Consurf server^28, 29^ and SASA values were used to evaluate Cis-1, Cis-2, and X-1 interfaces (Supplementary Fig. 5). The interfacial residues within 3 Å of the adjacent monomer were determined for each interface. Most of the residues in the Cis-1 interface are highly conserved; those located between the ECH and ECL2 are among the most conserved in the Cldn family (Fig. 5d), with an average conservation of the interacting residues in the ECH of ~ 62% and 98% for the ECL2. In addition, these residues are highly exposed with SASA values of 80 Å^2^. The Cis-1 interface also exhibits several interfacial residues between TM2 on one protomer with TM1 and TM4 on the adjacent protomer that are not well conserved. The X-1 interface had lower average conservation than Cis-1, with a conservation of ~ 62% in the interacting residues of the ECH and ~ 76% in ECL2, but presented comparable SASA values. Surprisingly, X-1 did not show any interfacial residues within 3 Å of the adjacent protomer between the TM domains. The conservation ratios of interacting residues in the Cis-2 interface were considerably lower compared to Cis-1: ~ 61% in the ECH and ~ 52% in the ECL1 residues. Cis-2 did have the largest number of interfacial residues between the TM domains, but they were poorly conserved (Supplementary Fig. 5). Consequently, we focused our efforts on the Cis-1 interface, which displayed better conservation at the potential interface site, and Cis-2 was not considered for further analysis in the context of this study.

### Cis-1 and X-1 are distinct interfaces with partial overlap

**Fig. 6:**
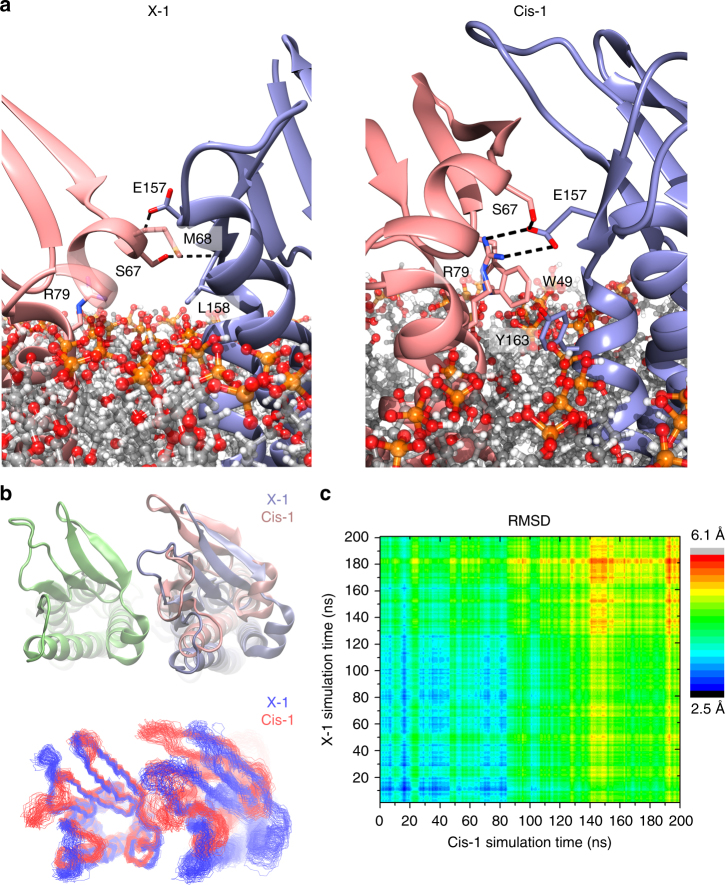
X-l and Cis-1 interfaces are independent and complementary. **a** Close views of X-1 and Cis-1 interfaces, where mCldn15 protomers are represented as cartoons and the side chains of residues participating in the corresponding interfaces are depicted as sticks (S67, M68, R79, E157, and L158) for X-1 and (W49, S67, R79, E157, and Y163) for Cis-1. Putative hydrogen bonds between Cldns are shown as black dashed lines. The relative location of the lipid bilayer is shown for clarification with lipids in stick representation. The heteroatoms were color coded as oxygen (red), nitrogen (blue), sulfur (yellow), aliphatic chains (gray), and phosphates (orange). **b** Molecular dynamics simulations of the X-1 and Cis-1 dimers. The initial interfaces (top, as cartoons) and ensemble traces (bottom) of X-l (blue) and Cis-1 (red) dimers generated over 200 ns, which show the range of thermal motions of each dimer. **c** 2D-RMSD plot of the superimposed X-1 and Cis-1 dimers over the 200 ns molecular dynamics simulations, RMSD values are color coded according to the scale bar shown

Analysis of the residue contacts between the ECH and ECL2 of adjacent Cldns in both Cis-1 and X-1 showed a degree of similarity between the interfaces. The X-1 interface features intermolecular hydrogen bonds involving the side chain of S67 residue with the backbone of L158 and the side chain of E157 with the backbone of M68 (Fig. 6). Residues S67 and E157 also participate in the Cis-1 interface but the intermolecular hydrogen bonding is formed between the side chains of residues S67 and E157. Cis-1 interface presents a distinct interacting region in which residue R79 side chain forms an additional intermolecular salt-bridge with E157. The interaction between residues R79 and E157 is not part of the X-1 interface. In the X-1 interface R79 is exposed, but it is too distant from the adjacent protomer to participate in intermolecular interactions (Fig. 6a).

The X-1 and Cis-1 interfaces could pivot around a common point of contact involving residues S67 and E157 to exchange into one another by simple Brownian dynamics. To examine the rotation compliance of the Cis-1 and X-1 interfaces, we compared 200 ns molecular dynamics simulations of each dimer configuration in a membrane environment. When several snapshots throughout the simulation for both dimers were aligned and overlaid, the conformational space of the dimers did not overlap (Fig. 6b). The structural divergence of the Cis-1 and X-1 dimers was quantified by measuring the RMSD between the X-1 and Cis-1 conformational ensembles, which increased over the course of the simulation (Fig. 6c). Taken together, our data suggests that X-1 and Cis-1 interfaces are two distinct *cis* conformations that present a common point of contact between the ECH and ECL2.

### Validation of Cis-1 interface by site-directed mutagenesis

**Fig. 7:**
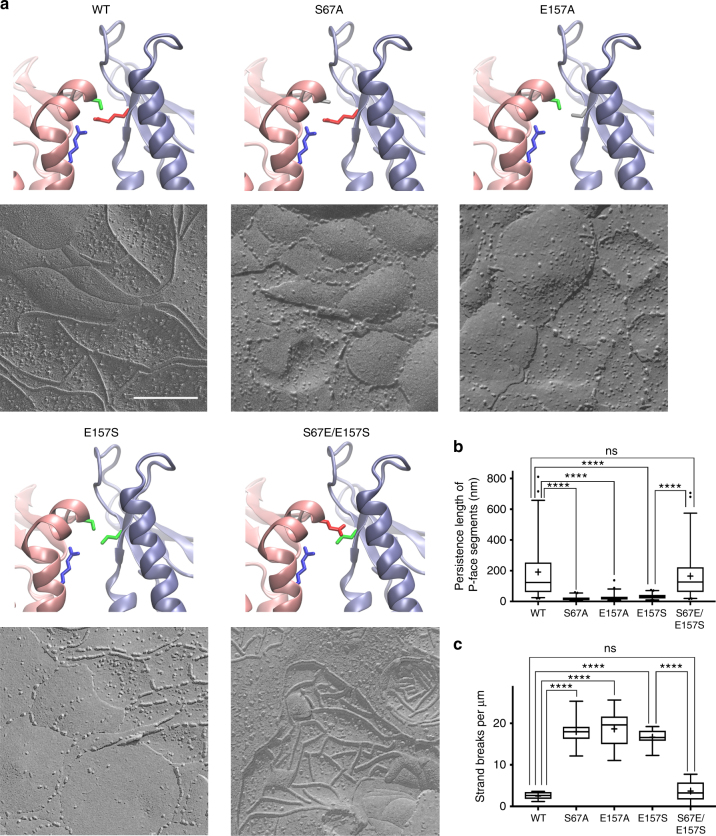
Freeze-fracture morphology of mCldn15 S67 and E157 mutants. **a** Cartoon representations of the Cis-1 interface in mCldn15 WT and mutants: S67A, E157A, E157S, and S67E/E157S. Residues at positions 67, 79, and 157 are color-coded green for serine, blue for arginine, red for glutamic acid, and gray for alanine. Freeze-fracture TEM of HEK293T expressing mCldn15 WT, S67A, E157A, E157S, and S67E/E157S showing the strand morphology. The P-face partitioning is distinctly altered in the S67A, E157A, and E157S. Size bar = 200 nm. **b** Quantification of P-face intramembrane strand persistence length (*n* = 50 segments) and number of strand interruptions per µm **c** (*n* = 10 strands) in WT mCldn15, S67A, E157A, E157S, and S67E/E157S shown as box plots: whiskers = 5th–95th percentile;+ = mean value; dots = outlier points, **** = *p* < 0.0001, ns = not significant

To validate the role of Cis-1 in tight junction strand formation, we performed a site-directed mutagenesis screen. We first examined the hydrogen bond forming residues S67 and E157, which are common to both X-1 and Cis-1 interfaces. Using fluorescence microscopy, we observed that the frequency of pairs of transfected cells that successfully formed tight junctions was 92% for mCldn15 WT and 87% for S67A mutant; however, it was reduced to 56% in the E157A mutant (Supplementary Fig. 4b). Freeze fracture of S67A and E157A mutants showed unusual partitioning of the intramembrane strands to both the P- and E-fracture faces at seemingly random intervals, giving the appearance of a discontinuous strand morphology (Fig. 7a). The persistence length in mCldn15 WT strands was 191 ± 184 nm, significantly higher (*P* < 0.0001) than the 18 ± 14 nm in S67A and 26 ± 23 nm in the E157A mutants (Fig. 7b, *n* = 50 for each). Although the number of discontinuities (Fig. 7c) in the WT was 2.5 ± 0.9 µm^–1^, it increased to 18.0 ± 3.3 in the S67A and to 18.6 ± 4.4 in the E157A (*P* < 0.0001, *n* = 10 strands each) likely due to weakening of the lateral interactions.

To further validate the potential interaction between the hydrophilic residue pair S67 and E157, we switched these two residues to generate an S67E/E157S double mutant and compared it with the E157S single mutant. Although the single E157S mutation produced a discontinuous strand morphology, the S67E/E157S double mutation restored the WT freeze-fracture phenotype (Fig. 7a). Also, the frequency of tight junction formation in pairs of transfected cells increased from 68% (*n* = 110) in the E157S single mutant to 89% in the S67E/E157S (*n* = 138) double mutant (Supplementary Fig. 4b). The measured persistence length in E157S was 32 ± 18 nm (Fig. 7b), whereas in the S67E/E157S double mutant was 164 ± 149 nm. Similarly, the E157S mutant (Fig. 7c) displayed an increased number of discontinuities (16.6 ± 2.1 µm^–1^) compared with the S67E/E157S double mutant (3.7 ± 2.5 µm^–1^). These data suggest that S67 and E157 residues from neighboring Cldns interact and participate in a cis interface.

**Fig. 8:**
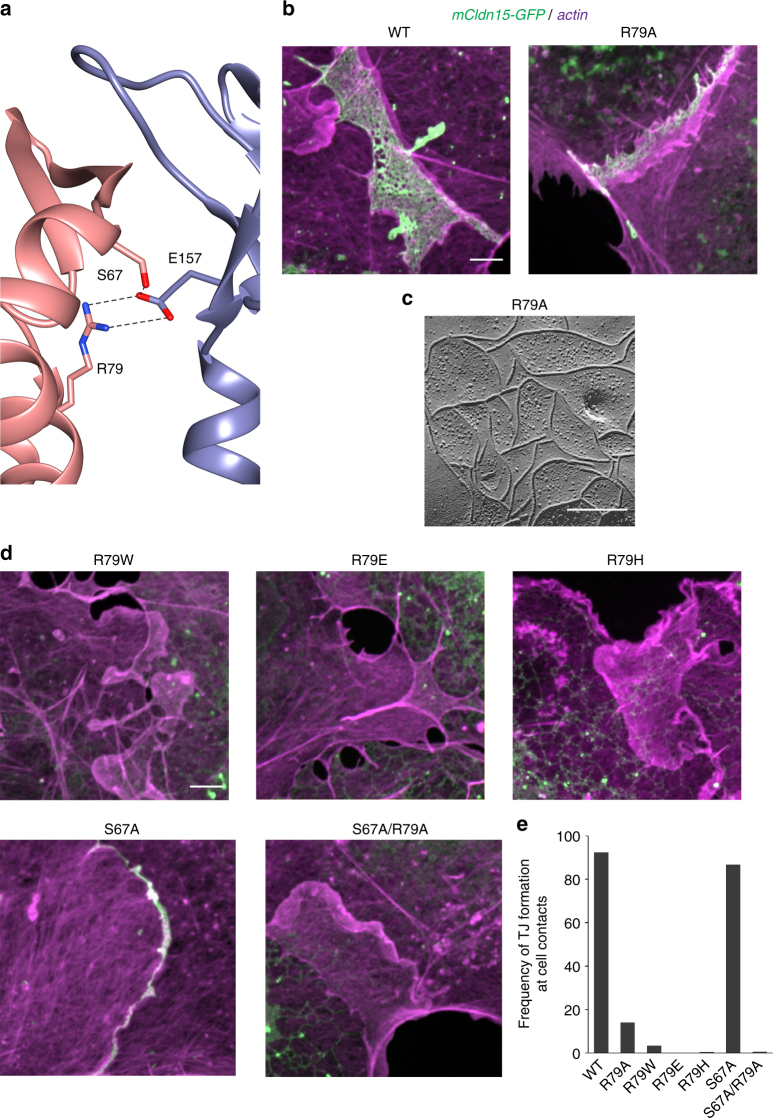
The Cis-1-specific residue R79 in mCldn15 is involved in strand formation. **a** Cartoon representations of the mCldn15 Cis-1 interface highlighting residues S67, E157, and R79, and showing putative hydrogen bond interactions (dashed lines). **b** Confocal imaging of COS7 cells expressing mCldn15-GFP WT or the R79A mutant (green). Cellular actin was counterstained with Alexa 568-phalloidin (shown in magenta). **c** Freeze-fracture morphology of tight junction strands in HEK293T cells expressing mCldn15 R79A. Scale bar = 200 nm. **d** Confocal imaging of COS7 cells expressing mCldn15 mutants R79W, R79E, R79H, S67A, and S67A/R79A. **e** Percentage of transfected cell pairs that form tight junction at cell contacts for the mCldn15 WT and mutants. Scale bars = 5 µm for all confocal images, WT *n* = 104, R79A *n* = 106, R79W *n* = 202, R79E *n* = 143, R79H *n* = 402, S67A *n* = 150, S67A/R79A *n* = 150

To validate the Cis-1 interface formed independently of the X-1 interface, we examined the Cis-1-specific residue R79. In the Cis-1 model, the most evident interactions of R79 are the pair of hydrogen bonds with residues S67 and E157 (Fig. 8a), but we cannot exclude additional interactions. The requirement of the R79 residue for strand formation was probed using a series of point mutations. The R79A mutant formed tight junctions with normal appearing morphology (Fig. 8b, c), whereas mutations of R79 to bulky (R79W and R79H) or negatively charged (R79E) residues prevented tight junction strand formation (Fig. 8d). Interestingly, R79A mutant formed tight junctions in only 14% of the transfected cell pairs compared with 92% in WT (Fig. 8e). We detected tight junction strands in only 3.5% of cell pairs transfected with R79W (Fig. 8e).

To determine whether R79 mutations affect Cldn trafficking and plasma membrane expression, we examined the mean fluorescence intensity of mCldn15-GFP at the edge of the cell, over lamellipodial, or filopodial regions of transiently transfected COS7 cells. These regions of the cell are commonly free of endoplasmic reticulum and vesicular organelles (Supplementary Fig. 6). The expression pattern of mCldn15-GFP WT and mutants at plasma membrane and endoplasmic reticulum varied broadly between transfected cells that form tight junctions (Supplementary Figs. 6 and 7a). R79 mutants showed on average equal or higher expression levels than WT (Supplementary Fig. 7b). These results suggest that plasma membrane expression levels of R79 mutants were not a limiting factor in tight junction strand formation.

Residues S67 and R79 participate in two of the three conserved interactions in Cis-1 but when individually mutated to alanine (S67A and R79A) do not prevent tight junction strands formation (Fig. 8). Interestingly, the S67A/R79A double mutant completely prevented tight junction formation (Fig. 8d, e), suggesting redundancy in their contributions to Cis-1. Our Cis-1 model suggested that S67 and R79 interact with E157 (Fig. 8a). However, as we showed above, E157A mutation alone does not completely abolish strand formation, suggesting that R79 may be involved in additional interactions other than with residues S67 and E157.

### A deafness mutation in Cldn14 maps to the Cis-1 interface

**Fig. 9:**
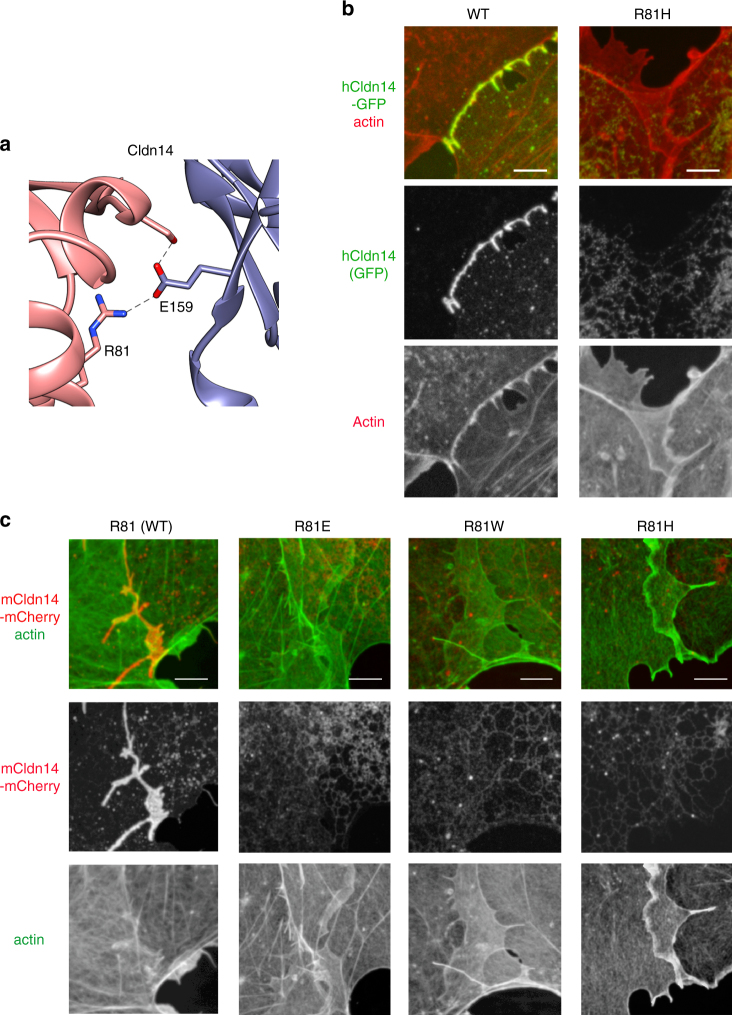
Deafness-related Cldn14 R81H mutant does not form tight junction strands. **a** Cartoon representation of the mCldn14 Cis-1 interface highlighting residues R81 and E159 and showing putative hydrogen bonds (dashed lines). **b** Confocal imaging of COS7 cells expressing hCldn14-GFP WT and R81H mutant (green) and counterstained for actin with Alexa 568-phalloidin (red). **c** Confocal imaging of COS7 cells expressing mCldn14-mCherry, R81E, R81W, and R81H (red), counterstained with Alexa 488-phalloidin (green). Scale bars = 5 µm

In order to further assess the biological relevance of the Cis-1, we searched the literature for human mutations in Cldns that map to residues involved in Cis-1. The missense mutation R81H in Cldn-14 (Cldn14) was reported to be associated with the DFNB29 form of human deafness^30, 31^. We examined human and mouse Cldn14 (hCldn14 and mCldn14) sequence alignment and homology models based on the mCldn15 crystal structure and found that the S67, R79, and E157 equivalents (S69, R81, and E159) were in similar structural positions to those in mCldn15 (Supplementary Fig. 8). To verify the importance of R81 in Cldn-14, we analyzed hCldn14 R81H mutant, matching the reported DFNB29 mutation, as well as the mCldn14 mutants R81H, R81E, and R81W. All mutant forms were clearly detected at the plasma membrane at the edges of COS7 cells and in filopodia using fluorescent mCherry tags (Supplementary Fig. 9), but failed to form tight junctions (Fig. 9). The expression of the R81 mutants in the plasma membrane was also examined by immunofluorescence labeling using an antibody specific to the mCldn14 C terminus^32^ and found to be equal or higher than the WT (Supplementary Fig. 10a, b).

We also tested tight junction formation when coexpressing mCldn14-GFP WT with mCldn14-mCherry R81H or hCldn14-mCherry WT with hCldn14-GFP R81H. In both cases we observed the tight junction formation by the WT Cldn, whereas the mutant Cldn was excluded from the tight junctions (Supplementary Fig. 11), suggesting that the R81H mutant does not exert a dominant-negative effect on the WT form, which is consistent with the recessive nature of DFNB29^30^.

## Discussion

Live imaging of cultured cells shows that Cldn strands undergo lateral movements and remodeling, which involves a range of arching and bending motions. Some of these movements appear to conform to the local membrane and cytoskeletal dynamics at the site of interaction between adjacent cells. Once the strands are formed, they keep a relatively stable length, suggesting that the strands tend to dynamically rearrange themselves rather than going through cycles of deconstruction and reformation. This lateral flexibility of the tight junction strands exhibited in the cell culture model is likely critical for maintaining the integrity of the tight junction barrier during epithelial and endothelial tissue remodeling.

Tight junction strands appear to have two distinct lateral flexibility modes: (i) arching, which extends uniformly over a segment of the strand, and (ii) bending, which has more abrupt curvature at discrete locations. These lateral flexibility modes should be an important feature of any model of tight junction molecular architecture. At the molecular level, strand curvature is presumably a result of Cldn protomer rotations within the linear Cldn oligomer. The estimated angles between protomers that are needed to account for the arching and bending of the mCldn15 strands range between 0–20°. In addition, in the case of a double row model^9^, bending would generate simultaneous tensile and compressive forces on opposing Cldn rows and require local complementary rotations.

In our experiments with mCldn15-transfected cells, the dynamic arching of Cldn strands are likely elastic deformations, where the strands evenly distribute small angles and displacements over many monomers. This type of elastic arching has been observed in other biological linear structures such as actin filaments^33, 34^. It is likely that the higher curvature bending exceeds the elastic arching of the tight junction strand, and that they involve some variability in the *cis* interactions between protomers. Although we do not expect that COS7 and HEK293T cells express native tight junction proteins, we cannot rule out the possibility that some of the Cldn bending behavior is influenced by other proteins.

There is no information available to date, regarding the rotation compliance of the X-1 interface^9^, which was largely based on the rigid crystal structure of mCldn15. Our molecular dynamics simulation of the X-1 dimer shows a limited thermal rotation of the protomers. The Cis-1 interface we identified has an ~ 17° rotation between Cldn monomers and could contribute to the structural variability required for strand flexibility. However, the mutations in Cis-1 residues either caused the strands to appear discontinuous in freeze-fracture or prevented the strand formation entirely. It is possible that structural variability and flexibility are inherent properties of tight junction strands where mutations affecting residues that impart flexibility prevent strand formation. Cldn strands may have multiple interfaces, similar to those observed in other oligomer systems^35^. Coarse grain molecular dynamics simulations of single row Cldn strand models exhibit a variety of monomer orientations, including Cis-1-like interactions^36, 37^. In a double row strand model^9^, the presence of Cis-1 would also require a complimentarily rotated Cis-interface.

Cis-1 involves highly conserved residues in the ECH and ECL2 of adjacent Cldn monomers. We observed that mutations of several individual residues (S67, M68, or E157) involved in the Cis-1 and/or X-1 interfaces did not prevent the strand formation, suggesting redundant interactions. However, the mCldn15 S67A and E157A mutants formed tight junction strands with altered freeze-fracture morphology, with intra-membrane strands partitioning randomly to the P- and E-fracture faces. It has been previously observed that Cldns can exhibit a range of freeze-fracture morphology^18, 38, 39^. For example, WT Cldn2, 5, and 14 have been shown to display a “discontinuous” freeze-fracture morphology^40–42^. However, there is only one previously reported mutant, Cldn-1 S53E/K65D, associated with a change in the P-/E-face freeze-fracture morphology^11^. The exact cause of the discontinuous freeze-fracture morphology is not well understood, but has been attributed to a variable degree of *Cis* oligomerization of the strand^43^. It has also been reported that mutations to Cldn3(E158R) and Cldn5 (E159Q), the equivalent of mCldn15 E157, prevent tight junction formation^8, 11^.

Our Cis-1 model predicts that R79 is involved in mCldn15 strand formation. This prediction is supported by the loss of strand formation when R79 is mutated to R79E, R79W, or R79H, and the low frequency of tight junction formation in the R79A mutant. We also show that these mutant proteins are targeted to the plasma membrane by fluorescence imaging of the cell edges and filopodial protrusions. Filopodia are typically devoid of vesicles or endoplasmic reticulum and the fluorescence detected should be from mCldn15 in the plasma membrane. Quantification of the R79 mutants showed plasma membrane expression levels equal or greater levels than the mCldn15 WT excluding the possibility that tight junction strand formation was impaired by reduced plasma membrane expression. Furthermore, the normal appearance of the mCldn15 R79A tight junction strands suggests that the mCldn15 monomer structure was not aberrantly affected by the mutation.

The functional relevance of Cis-1 and R79 is further supported by the finding that hCldn14 R81, the equivalent to mCldn15 R79, is associated with DFNB29 recessive deafness^30, 31^. We now show that hCldn14 R81H is unable to form tight junction strands. In addition, we observed normal tight junctions when co-expressing WT hCldn14 and hCldn14 R81H, demonstrating that the mutant does not have a dominant-negative effect, which is consistent with the recessive nature of DFNB29^30^. In the cochlea, Cldn-14 strands form the most apical part of the tight junction network between hair cells and supporting cells^32^, acting as the first selective ion barrier to the high potassium content of the endolymph. The loss of these strands likely causes a loss of proper ionic composition around the basolateral surface of hair cells, leading to the loss of hair cells and deafness, as shown in the Cldn14 knockout mouse^44^.

In addition to arching and various degrees of bending, tight junction strands exhibit other structural variability including various branching shapes and possibly strand forking, all requiring compliance and/or variability in the *cis* interface. Cis-1 or X-1 alone are not likely to be sufficient to explain the range of tight junction strand branching angles. Also, mutations of the X-1 and Cis-1 common residues S67 and E157 did not prevent the strand branching. Our HADDOCK modeling provided additional candidates for *cis* interface (Cis-2 and Cis-3 interfaces). Cis-2 and Cis-3 can form approximate 60° and 90° angles with a parent strand, respectively, which could account for higher branching angles. Their validity, natural occurrence, and potential involvement in tight junction strand branching have not been examined in this study. Possible applications of this structural variability are endless and may involve multiple forms of *cis* and *trans* interactions, and may depend on both Cldns and other cell-specific factors^18, 40^. How these alternative interactions cooperate to form the natural configuration of the tight junction strands remains to be determined. A better understanding of tight junction strand molecular architecture will certainly result from future investigations on the cooperativity between *cis* and *trans* interactions that interlocks the protomers into a linear flexible strand. Perhaps a biologically intriguing question one can ask from our findings is how such flexible and variable structure of tight junction strands is able to maintain and regulate its selective ion permeability function.

## Methods

### Construction of claudin monomers and strands

The model of the mouse mCldn15 monomer, constituted by residues 1–186, was generated using the monomeric crystal structure of mCldn15 (PDB ID: 4P79)^6^ as template. The mutated palmitoylation sites and missing residues at the ECL1 (Val34 to Thr41) were filled from the SEQRES records in the PDB file. The missing ECL1 residues were modeled using the homology modeling module available within Schrodinger software package^45^ using the crystal structure of the Cldn19/CPE complex as a template (PDB ID: 3X29)^46^. The resulting structure was used as an initial input into the rest of computational methods used in this work. Homology models of mCldn14 (AAG60051.2) and hCldn14 (AAG60052.1) were generated using the SWISS-MODEL Server^47^ and mCldn15 (PDB ID: 4P79) as the template structure. The models were superimposed using UCSF-Chimera^48^. Sequence alignments of mCldn15, mCldn14, and hCldn14 were generated using the T-Coffee webserver^49^.

The initial 16mer model of claudin consisting of 16 protomers was built by adding four extra protomers to Suzuki’s 12mer model^9^. The added protomers were positioned using the protomer-protomer distance restrains extracted from Suzuki’s model. The initial 16mer model was used to obtained two cis-8mers that were separated 4 nm from each other to avoid clashes within the additional loop residues. The new 16mer was then rebuilt by restraining the ɑ-carbons of each 8mer onto the initial 16mer, while the loops were relaxed throughout a 10,000 minimization steps. The system was subjected to 1 ns molecular dynamics simulation in which the intermolecular hydrogen bonds were maintained by distance constraints between the donor and acceptor of each hydrogen bond, and positional restraints were applied to ɑ-carbons. An extra 1 ns simulation was further performed to remove the ɑ-carbon constraints, while maintaining the hydrogen bond restraints. During this protocol, the ECLs were re-positioned and minimized to avoid the clashes. The 8mer strand model was built with similar protocol in which four boundary protomers were removed

### Coevolutionary coupling and conservation ratio analysis

The conservation ratio of Cldn residues and co-evolutionary coupled mutants between pairs of residues in a single Cldn protomer were calculated using Consurf (http://consurf.tau.ac.il)^28, 29^ and EVfold servers respectively (http://www.EVfold.org)^23, 24, 26, 50^. In the case of Consurf the default parameters were used, whereas in EVfold the helical TM segments were defined as in uniprot (Q9Z0S5) and a pseudo-likelihood maximization approach was used as a coupling scoring function.

To only select sequences of vertebrate Cldns we used a 30% sequence identity cutoff. The resulting 1042 sequences (sequences/alignment length = 5.56) were aligned using jackhmmer-algorithm to generate the multiple sequence alignment. The top 500 pairs with the highest EVcomplex score were selected (Evcomplex score > 0.47), out of the 19,115 pairs identified. Those pairs involving C-terminal residues (187–227) not available in the crystal structure were excluded. The final pairs were further evaluated by computing the distance between the center of mass of the side chains of each pair; the distances < 10 Å were included in the protein contact map of the mCldn15 monomer using CMView^51^. Approximately 71% of the ECs in the final set (375 ECs with coupling strength ≥ 0.1) overlapped with the contact map and were considered to reflect the Cldn monomer folding information. The distance distribution showed that the rest of these pairs (109 pairs) have distances between the center of mass of the residues involved longer than 20 Å. The majority of the coupling pairs are in close proximity only in a protomer context and, as a consequence, they can be considered as potentially contacts present in Cldn–Cldn interfaces. These selected pairs were further mapped on to the interfaces of Cldn dimers obtained from the protein–protein docking procedure (see below).

### Interface search and claudin dimer modeling using HADDOCK

To search the potential protomer interfaces between Cldns, we performed automated molecular docking of Cldns with the program HADDOCK 2.1^22^. The Averaged structure of mCldn15 monomer from the last 10 ns molecular dynamics simulation was used as input for each of the docking runs. HADDOCK can use different types of information to guide the docking process (ambiguous interaction restraints), which can be defined as active and passive. For example, residues that are thought to be stabilizing interactions in the dimer interface are defined as “active,” whereas those in the proximity are considered as “passive.” As a first approximation, we used the so called “blind” docking method in which all residues with both high exposure and high conservation ratio were input as “active” residues. The degree of exposure for each of the first 186 residues of the mCldn15 monomer was obtained from the molecular dynamics simulation (described below) by SASA using a water molecule as a probe (1.4 Å radius). We defined “active residues” as all residues with SASA > median value (56.7 Å^2^). The neighboring residues of those “active residues” were selected as “passive” for HADDOCK input in order to consider all the exposed residues in the docking run. We generated 1000 poses and the best 200 HADDOCK models of Cldn dimers were clustered. As a result, 13 clusters were identified. The final set of poses was constituted by the representative clusters with tilt angles less than 30° between the two Cldn protomers to ensure that the orientation of the two protomers was compatible with the presence of a lipid bilayer.

### Molecular dynamics simulations of claudin assemblies

The N- and C-terminal domains of mCldn15 were completed as NH_3_^+^ and COO^–^, respectively. The monomer and oligomer structures of mCldn15 were energy-minimized by fixing the positions of the backbone heavy atoms. To mimic the environment of Cldn strands between adjacent cells, the double row mCldn15 oligomers with 8 protomers (8mer) and 16 protomers (16mer) were inserted into opposing POPC bilayers using the CHARMM-GUI membrane builder generator^52^. To construct the two-bilayer system, each of the double-strands in the 8mer and 16mer were inserted in lipid bilayers separately and then superimposed on the strand. Both bilayers were combined to form the two-membrane layers/claudin strand system. One bilayer of POPC was used in the case of the monomer and *cis*-dimers. POPC lipids were randomly selected from a lipid library and assembled around the strands to satisfy a lipid surface area of ~ 68.3 Å^2^ at 310 K^53^. The simulation systems were solvated by water molecules (TIP3P, three-site transferrable intermolecular potential water model), and sodium and chloride ions were added to neutralize the system, at a total concentration of ~ 150 mM. The systems were minimized for 5000 conjugate gradient steps, with the protein backbone and lipid head group atoms fixed followed by additional 5000 conjugate gradient minimization steps without the protein backbone or lipid head groups fixed. In the equilibration run, each system was gradually relaxed by a series of dynamic cycles, in which the harmonic restraints on the proteins and lipid head groups were gradually released to optimize the protein–water, protein–lipids, and water–lipids interactions. In the production stage, NPAT (constant Number of atoms, Pressure, surface Area, and Temperature) ensemble at 310 K was used. The surface area in the *xy* plane (membrane plane) remained constant with a volume change in the *z* direction. A switch function with a twin-range cutoff at 12 and 14 Å was used to evaluate the van der Waals interactions. Particle mesh Ewald method was used for long-range electrostatic interactions evaluation. Each simulation system was run twice using the same strand configuration, with different lipid conformations randomly selected from the lipid library and different initial velocities for all atoms. All molecular dynamics simulations were performed using the NAMD software^54^ with CHARMM27 force field^55^. Molecular dynamics trajectories were saved every 2 ps for analysis.

### Analysis of molecular dynamics simulation results

The RMSDs of the ɑ-carbons with respect to the initial minimized crystal structure were used to evaluate the relative structural stability of the oligomers throughout the simulations. SASA of individual residues of mCldn15 monomer was evaluated using a probe with a radius of 1.4 Å during the last 20 ns molecular dynamics trajectories.

To evaluate the relative structural stability of Cldn–Cldn interfaces, the trajectory for each system was extracted from the last 20 ns of explicit solvent molecular dynamics without water molecules and ions. The solvation energies of all systems were calculated using the generalized Born method with molecular volume (GBMV)^56^ after 500 steps of energy minimization to relax the local geometries caused by the thermal fluctuations that occurred in the molecular dynamics simulations. In the GBMV calculation, the dielectric constant of water was set to 80 and a symmetrical distribution of dielectric constants inside lipid bilayers was used with no distance cutoff.

### Expression vectors, mutagenesis, and cell transfection

mCldn15 complementary DNA cloned in the pCMV6-AC-GFP mammalian expression vector was purchased from Origene (Rockville, MD, USA), hCldn14 cDNA was purchased from Genecopoeia (Rockville, MD), and the mCldn14 cDNA was derived from Nunes et al.^32^. Site-directed mutagenesis was performed using the KAPA HiFi PCR kit (Kapa Biosystems, Inc., Wilmington, MA, USA) or the Agilent Quickchange Lighting kit (Agilent, Santa Clara, CA). All cell lines used in this study were originally obtained from American Type Culture Collection. COS7 or HEK293T cells were seeded in 35 or 60 mm cell culture plates and cultured in Dulbecco’s modified Eagle medium (Invitrogen) supplemented with 10% fetal bovine serum. Cells were transfected with plasmids encoding for WT or mutant Cldn using Lipofectamine LTX (Life Technologies, Carlsbad, CA) and the cells were allowed to express the protein for at least 24 h. Each transfection and subsequent analysis was carried out at least twice.

### Immunocytochemistry and live-cell imaging

Transfected COS7 were fixed with 4% paraformaldehyde in phosphate-buffered saline (PBS) for 20 min and counterstained with 17 nM Alexa Fluor® 405-, 488-, or 568-phalloidin (Biotium, Fremont, CA) in 0.5% Triton X-100 for 20 min. Immunocytochemistry for mCldn14-mCherry was performed using the custom-made rabbit polyclonal antibody PB321^32^ at a concentration of 1.7 µg mL^–1^. Imaging of the samples was carried out using a confocal laser-scanning microscope (Zeiss LSM 780 Meta, Zeiss, Germany) or a Nikon TI system equipped with a spinning disk confocal head, a 3.4 secondary magnification for improved dynamic range^57^, and a high performance electron-multiplying charge-coupled device (CCD) (Andor Ultra 888 or 897); images were acquired and analyzed using NIS-Elements. Live-cell imaging was performed on Rat1 cells stably expressing mCldn2-GFP^17^ seeded on collagen-coated glass-bottom dishes; imaging was performed using an incubator attachment on the Nikon system described above. Image brightness and contrast were uniformly rescaled across the entire image using Adobe Photoshop. For images demonstrating Cldn plasma membrane expression, the dynamic range and γ-level were uniformly adjusted across the entire image to allow simultaneous viewing of distinct endoplasmic reticulum and plasma membrane fluorescence levels.

Cldn membrane expression levels were measured as the mean fluorescence intensity of either the direct mCldn15-GFP fluorescence or by indirect immunofluorescence (PB321 primary antibody and goat anti-rabbit AlexaFluor® 488 secondary antibody) over randomly selected area of thin, lamellipodial, and filopodial extensions at the edge of Cldn-transfected COS7 cells. These very thin distal regions of the cell are characteristically free of endoplasmic reticulum and vesicular organelles. We were able to detect the low-level fluorescent signal of the fluorophores at the plasma membrane compared with that of the endoplasmic reticulum because of the high sensitivity and dynamic range of our spinning disk imaging system^57^. Expression level comparisons were made using identical acquisition settings. Similar areas of background and non-transfected cells were quantified as fluorescence intensity reference. A minimum of 50 membrane patches were measured from multiple cells and fields of view for each Cldn tested.

The frequency of tight junction formation was determined by quantifying the percentage of transfected cell pairs (*n* > 100) that formed tight junctions.

### Freeze-fracture electron microscopy

HEK293T and COS7 cells were prepared for freeze fracture as previously described^58^. Cells were fixed in 2% glutaraldehyde in PBS for 1 h and slowly transitioned to 30% glycerol as cryoprotectant. Cells were lifted with a cell scraper followed by “slam-freezing” using a LifeCell CF-100 equipped with sapphire disk cooled to − 186 °C as the freezing surface. Samples were fractured using a Balzer freeze-fracture apparatus and the replicas created by deposition of platinum at 45° followed carbon at 90° angles relative to the sample. The replicas were imaged using a JEOL 2100 TEM operating at 200 kV with a Gatan Orius 832 CCD. The SerialEM/Etomo software suite^59^ was used to both collect and analyze the data. At least two independent replicas were analyzed for every Cldn mutant. P-face persistence length was determined by measuring the length of 50 P-face segments per Cldn. Strand breaks were quantified by tracing 0.8–1.5 µm lengths of 10 randomly selected strands from at least 2 replicas for each condition (*n* = 10) and counting the number of disruptions in strand P-face segregation along the length of the strand.

### Curvature and angle analysis

For curvature analysis, 16 randomly selected mCldn15 tight junction strands were segmented from freeze-fracture images and exported to *xy* coordinates in FIJI. The radius of curvature was determined by fitting osculating circles using the arcfit method^19^ using 3-point sampling at 10 pixel intervals (~ 12 nm between points) along the length of curves. The curvature parameter *κ* was determined by taking the reciprocal of the radius of curvature. The angle per monomer step was estimated using the radius of curvature and the mCldn15 monomer centroid to centroid spacing of 2.8 nm observed in PDB ID:4P79. Strand branch angles were determined by measuring the acute angle between the two Cldn strands at the point of intersection.

### Statistical analysis

Cldn strand analysis sample sizes were estimated based on preliminary data and prior experience. Values reported are mean ± SD, error bars shown as the first SD unless otherwise stated. The significance of the freeze-fracture strand discontinuity and Cldn membrane fluorescence was determined by analysis of variance and the Tukey’s multiple comparison test in Prism (Graphpad Software, La Jolla, CA); *P*-values < 0.05 were considered significant.

### Data availability

All relevant data in support of the findings of this study are available from the corresponding authors by reasonable request.

## Electronic supplementary material

Supplementary Information

Description of Additional Supplementary Files

Supplementary Movie 1
