# Supplementary material for: Multiple claudin–claudin cis interfaces are required for tight junction strand formation and inherent flexibility
Source: Commun Biol. 2018 May 17;1:50. doi: 10.1038/s42003-018-0051-5 (PMC6123731; doi:10.1038/s42003-018-0051-5)
Supplement: Supplementary file 1 — Supplementary Information [file 42003_2018_51_MOESM1_ESM.pdf]

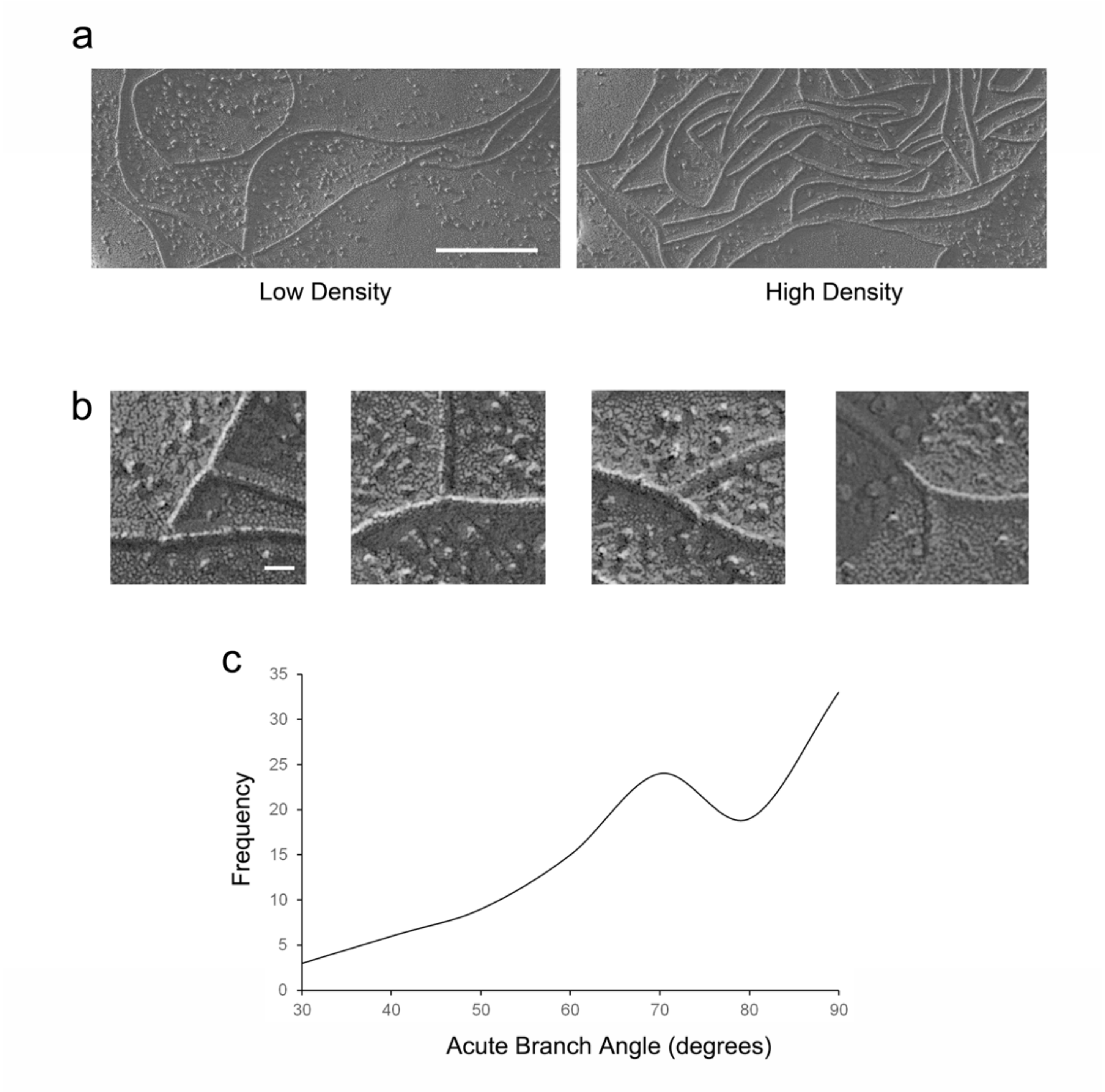

Supplementary Figure 1. TJ strand branching and bending in sparse and dense TJ networks. **a** Freeze-fracture TEM of HEK293 cells expressing mCldn15 displaying sparse (left image) and dense (right image) TJ networks. Scale bar = 200 nm. **b** Representative examples of branching morphologies observed in mCldn15 TJ networks. Scale bar = 20 nm. **c** Frequency distribution of strand branching angles (n=107).

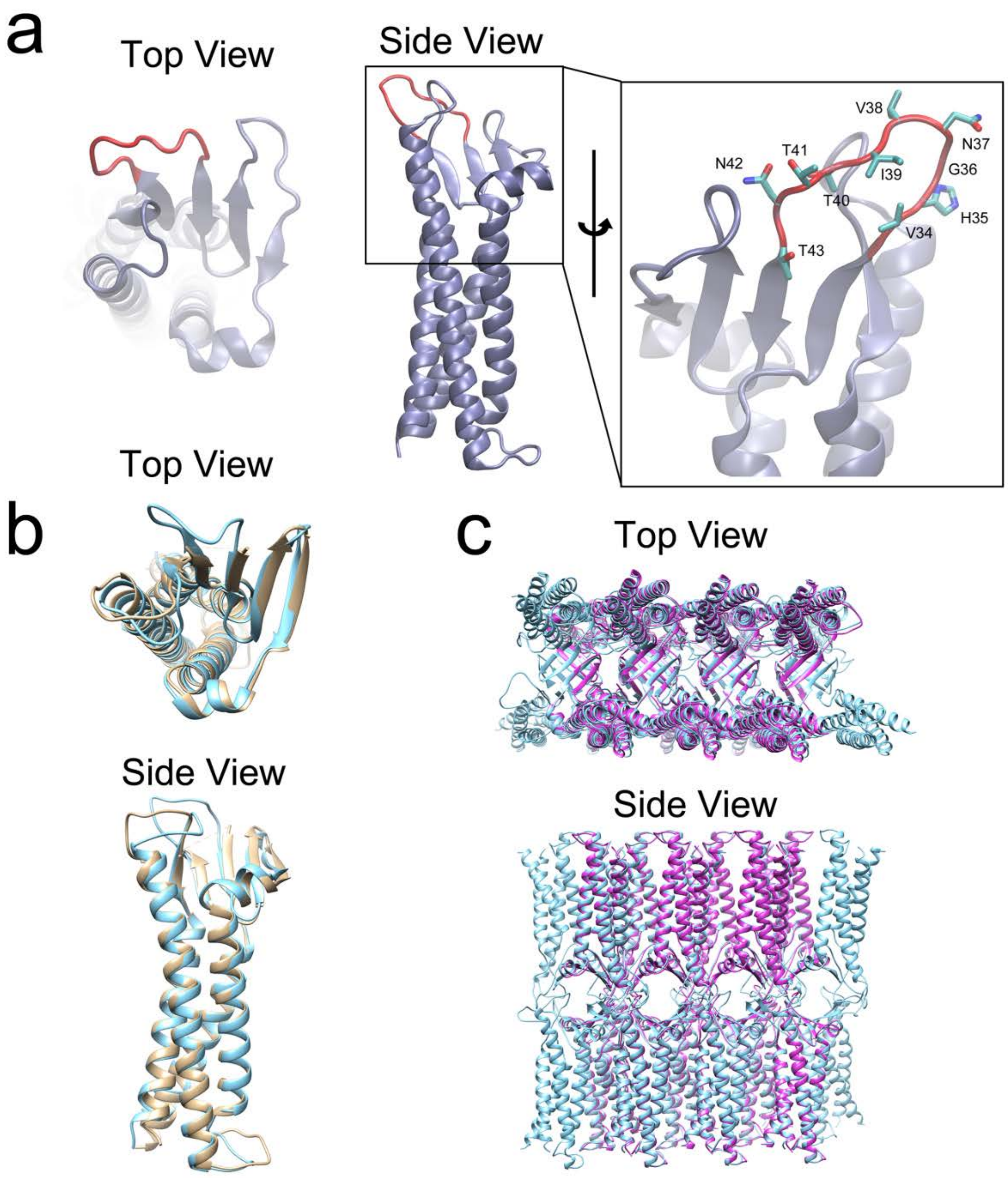

Supplementary Figure 2. Structure of mCldn15 model including residues V34-T41. **a** Top and side views of mCldn15 structure in cartoon representation with the modeled ECL1 residues V34-T41 highlighted in red. Residues V34-T41 side chains are shown in stick representation in the closeup view. **b** Overlaid structures of the modeled mCldn15 monomer (blue) and the mCldn15 crystal structure (tan, PDB ID:4P79). **c** Overlaid structures of our proposed 16mer model (blue) and the model proposed by Suzuki et al (purple).

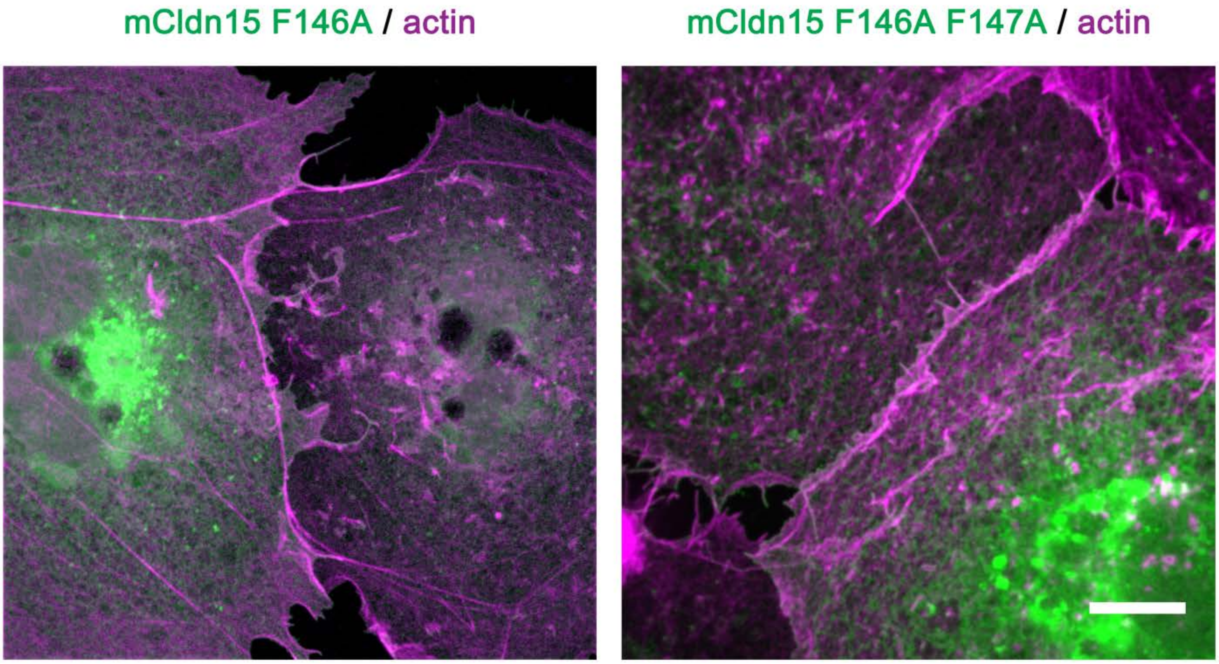

Supplementary Figure 3. mCldn15 F146 and F147 are essential for TJ strand formation. Confocal images of COS7 cells expressing mCldn15-GFP mutants: F146A and F146A/F147A (green) and counterstained for actin with phalloidin (magenta). No accumulation of mCldn15-GFP indicative of strand formation was observed at cell-cell contacts. Scale bar = 5 μm.

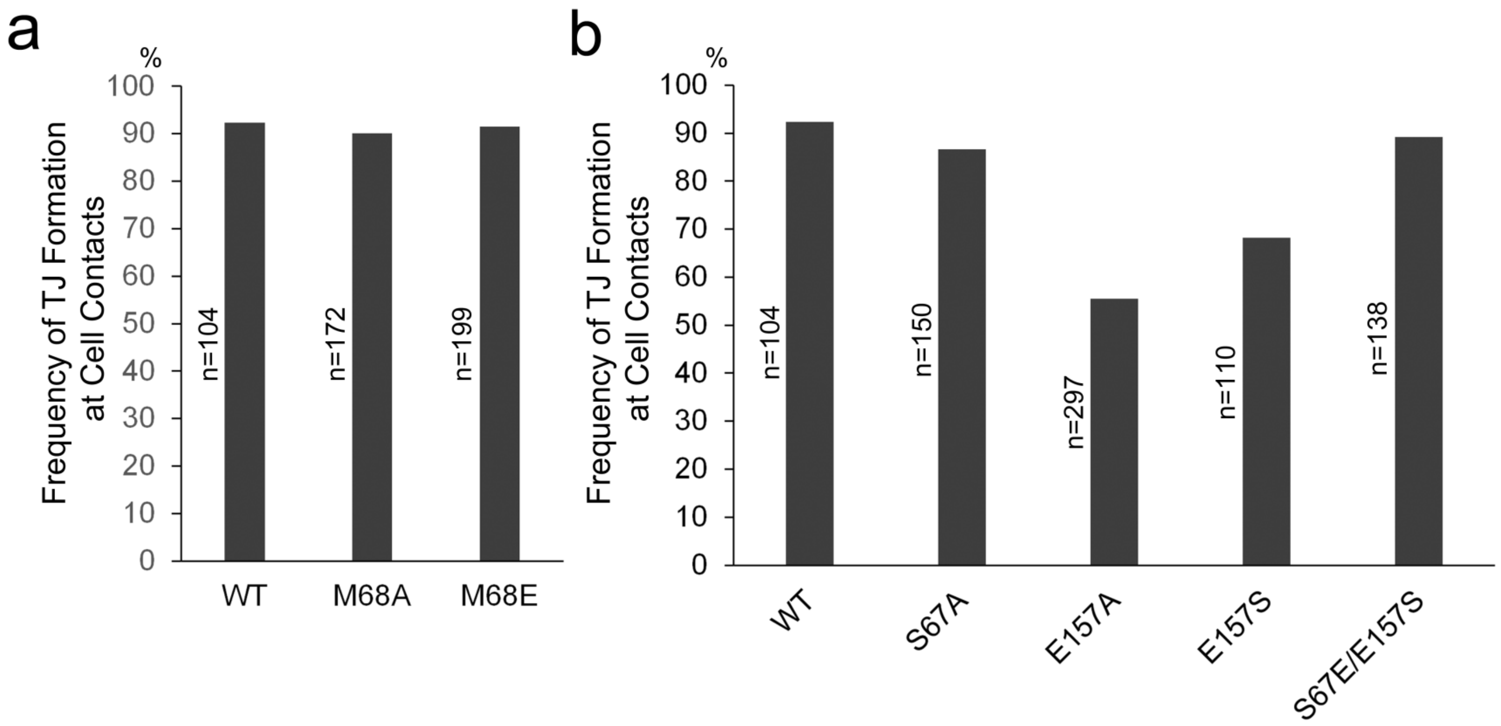

Supplementary Figure 4. Frequency of Cldn15 TJ formation between transfected COS7 cell pairs. Bar plots of the frequency of TJ formation between transfected COS7 cell pairs for **a** mCldn15-GFP WT, M68A, M68E and **b** S67A, E157A, E157S, and S67E/E157S. The number of cell pairs counted for each Cldn (n) is shown next to each bar plot.

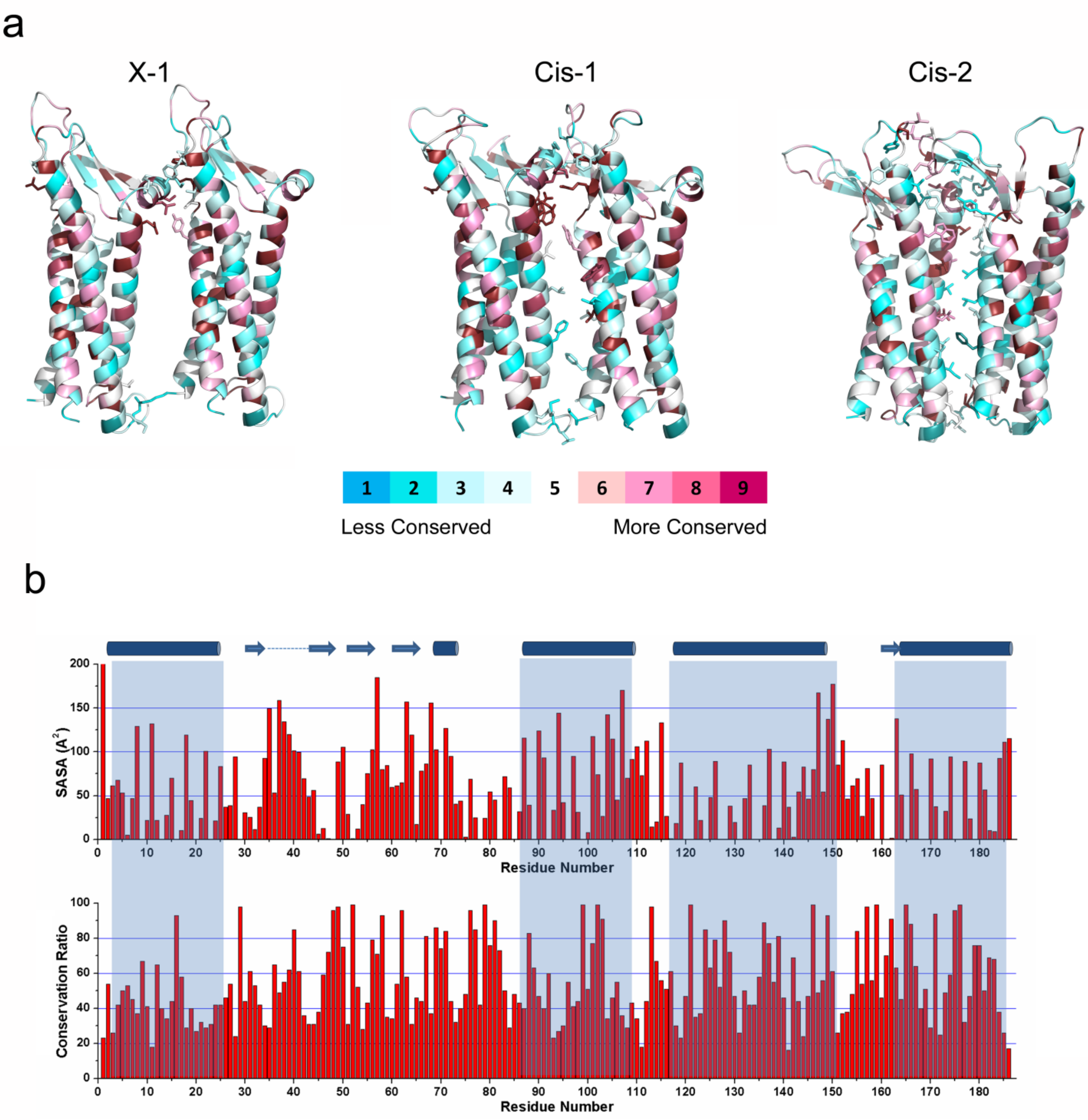

Supplementary Figure 5. Conservation and SASA of the X-1, Cis-1, and Cis-2 interfaces. **a** The conservation scores for each residue of mCldn15 mapped onto the Cis-1, Cis-2, and X-1 interfaces. Interfacial residues within 3 Å of the adjacent monomer are shown as sticks and colored by their conservation ratio as indicated by the scale bar. **b** Plot of SASA values for mCldn15 residues 1-186 and conservation ratio of mCldn15 residues compared to other Cldns. Secondary structure elements are shown on the top representing α-helix as cylinders and β-strands as arrows.

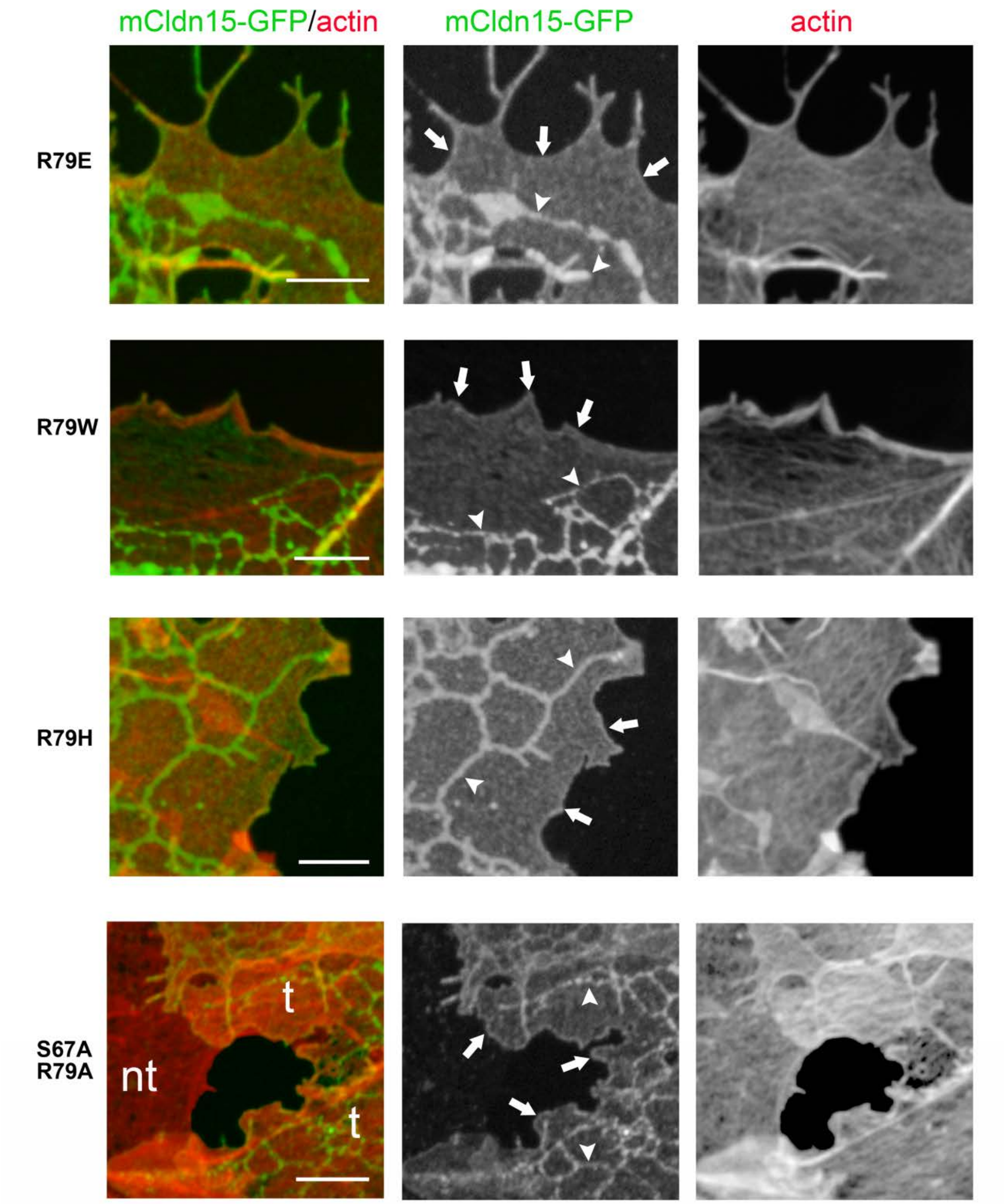

Supplementary Figure 6. Plasma membrane expression of mCldn15 mutants. Confocal images of COS7 cells expressing mCldn15-GFP R79 mutants (green) counterstained for actin with phalloidin (red). Cells transfected (t) with mCldn15 R79E, R79W, R79H, and S67A/R79A mutants showed expression of the mCldn15 throughout the entire cell surface, extending to the outer edges of the cells (arrows) in addition to the ER (arrowheads). Fluorescence was not detected in neighboring non-transfected cells (nt). Scale bars = 5 µm.

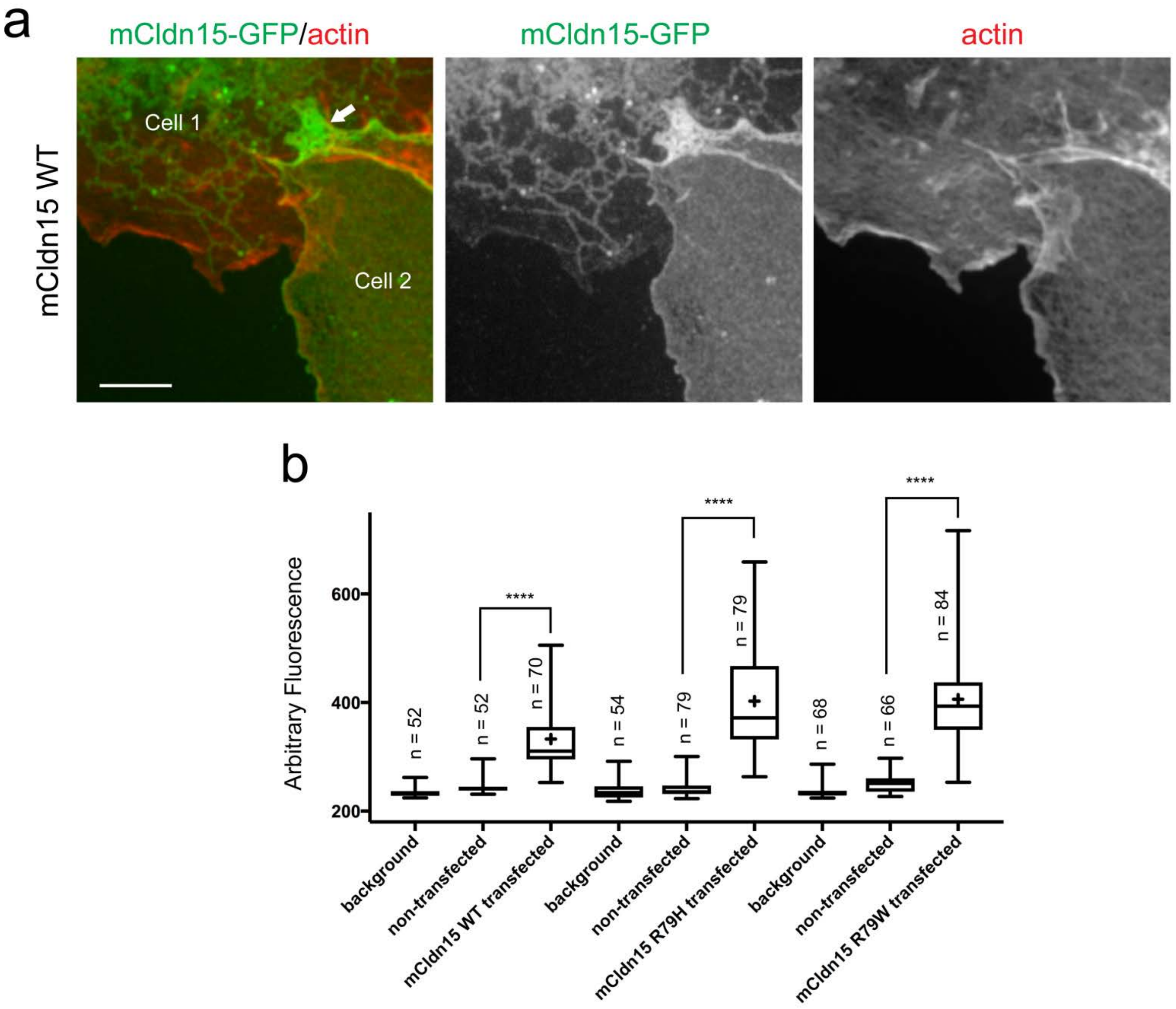

Supplementary Figure 7. Variability of mCldn15 plasma membrane expression. **a** Confocal images of two adjoining COS7 cells expressing mCldn15-GFP WT (green), the cells are counterstained with Alexa 568-phalloidin (red). Two cells (cell 1 and cell 2) forming a TJ (arrow) show broadly differing levels of mCldn15-GFP plasma membrane expression. Scale bar = 5 µm. **b** Box plot of mCldn15-GFP plasma membrane expression determined by measuring the mean fluorescence intensity over patches of plasma membrane at thin endoplasmic reticulum-free lamellipodial extensions. The mean fluorescence intensity of the background and over non-transfected COS7 are shown for comparison, Whiskers = min-max, + = mean value, number of fields (n) are indicated, **** = P<0.0001

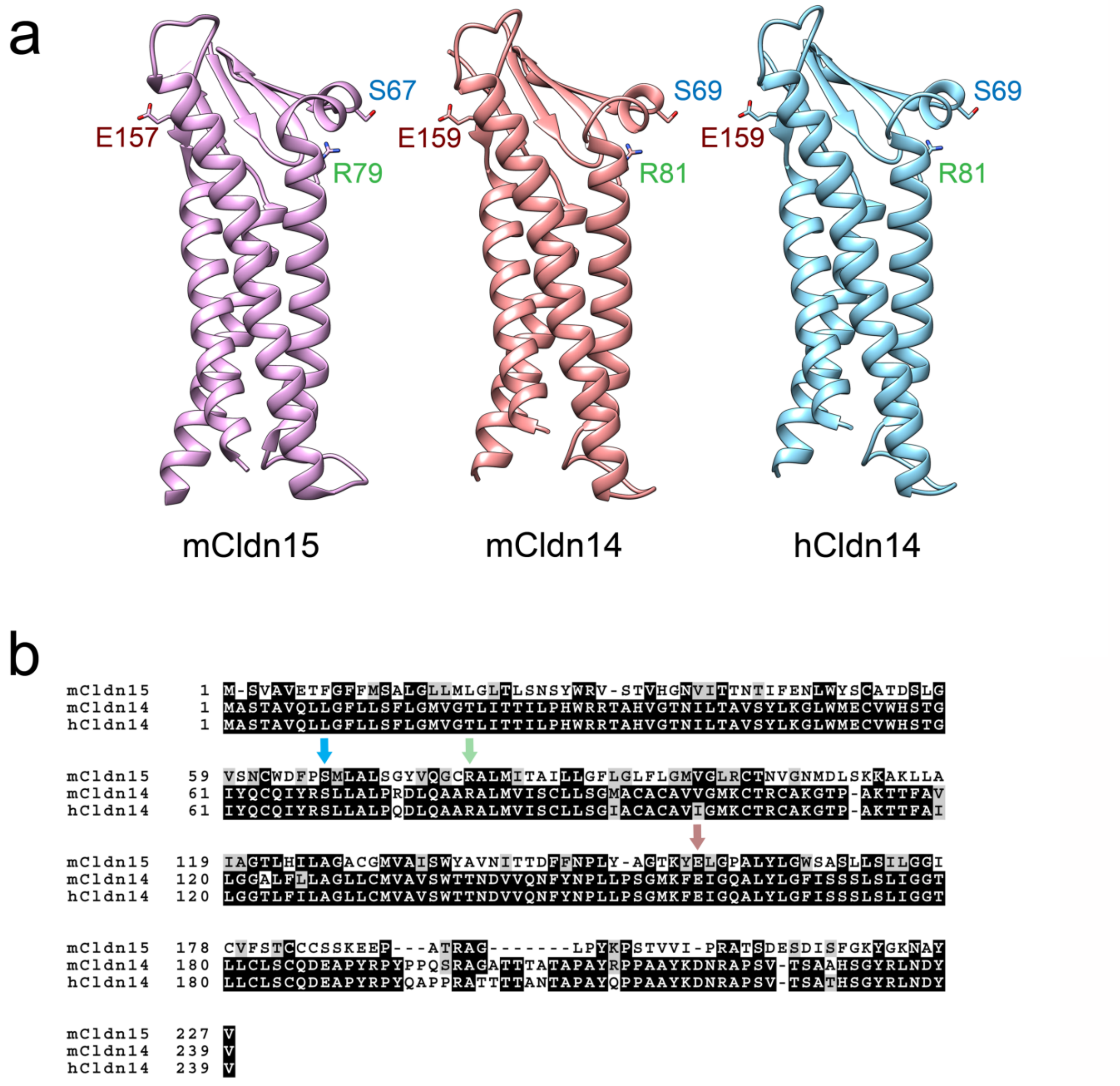

Supplementary Figure 8. Residues involved in Cis-1 interface are conserved in mCldn14 and hCldn14. **a** mCldn15 (purple) crystal structure (PDB ID: 4P79) was used as template to model the structures of mCldn14 (red) and hCldn14 (blue). The models obtained were structurally superimposed to the template. Side chains of key residues in the Cis-1 interface are shown as sticks. **b** Alignment of mCldn15, mCld14, and hCldn14 amino acid sequences; arrows indicate the positions of the residues involved in the Cis-1 interface: S67 (blue), R79 (green), and E157 (brown) in mCldn15. Conservation ratios are shown as black (100% conserved), gray (>50% conserved), and white (<50% conserved).

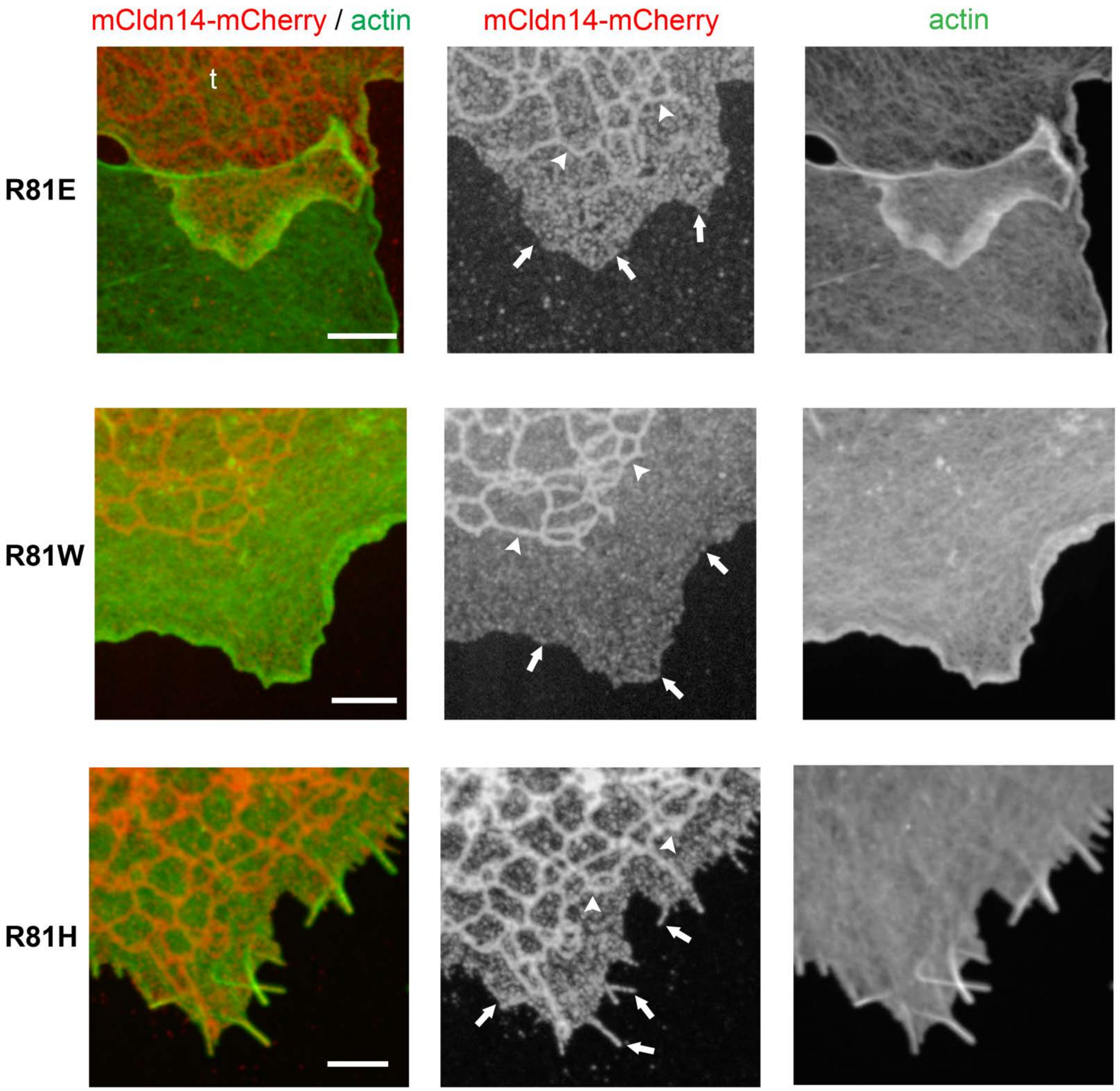

Supplementary Figure 9. Plasma membrane expression of mCldn14 mutants. Fluorescence confocal images of very thin lamellipodial extensions of COS7 cells expressing mCldn14-mCherry mutants and counterstained for actin with Alexa 488-phalloidin (green). mCherry fluorescence at the edges of the cell and filopodia (arrows) corresponding to the plasma membrane is observed for the R81E, R81W and R81H mutants. Cldn fluorescent signal over the plasma membrane and endoplasmic reticulum (arrowheads) was only observed in transfected cells (t), and absent in neighboring non-transfected cells (nt). Scale bar = 5 µm.

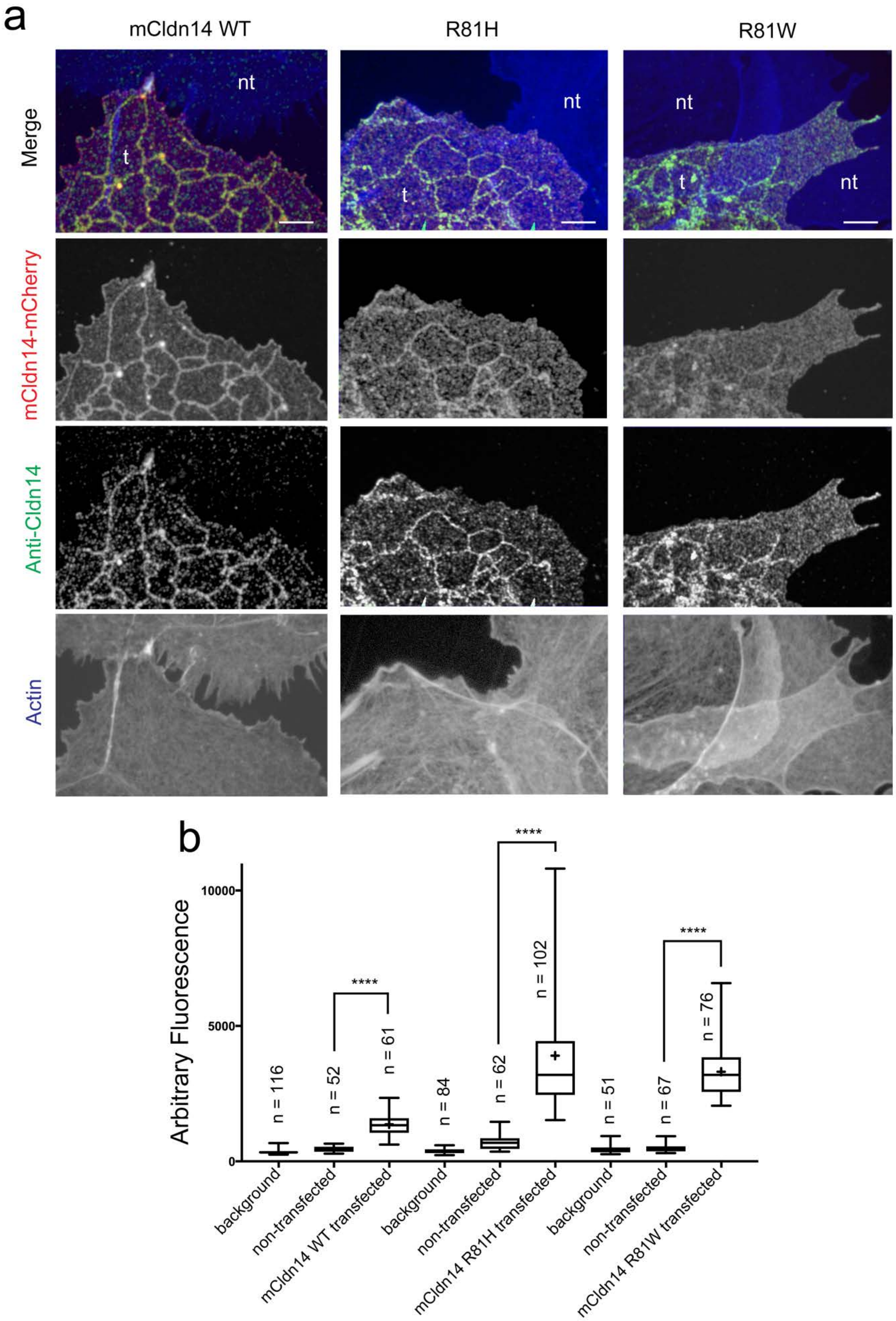
a
mCldn14 WT
R81H
R81W
Merge
mCldn14-mCherry
Anti-Cldn14
Actin
nt
t
b
Arbitrary Fluorescence
10000
5000
0
****
n = 116
n = 52
n = 61
n = 84
n = 62
n = 102
n = 51
n = 67
n = 76
background
non-transfected
mCldn14 WT transfected
background
non-transfected
mCldn14 R81H transfected
background
non-transfected
mCldn14 R81W transfected

Supplementary Figure 10 Quantification of mCldn14 plasma membrane expression. **a** COS7 cells transfected with mCldn14-mCherry WT, R81H, or R81W (red) and immunolabeled with anti-Cldn14 (green). Cells are counterstained for actin with Alexa 405-phalloidin (blue). Each field of view shows transfected (t) and non-transfected (nt) cells. Scale bars = 5 µm. **b** Box plot of the mean immunofluorescence intensity for Cldn14 over randomly selected endoplasmic reticulum-free areas of lamellipodial extensions. The mean fluorescence of the background, non-transfected and transfected COS7 cells are shown for comparison. Whiskers = min-max, + = mean value, the number of fields (n) measured is shown in the graph. **** = $P<0.0001$

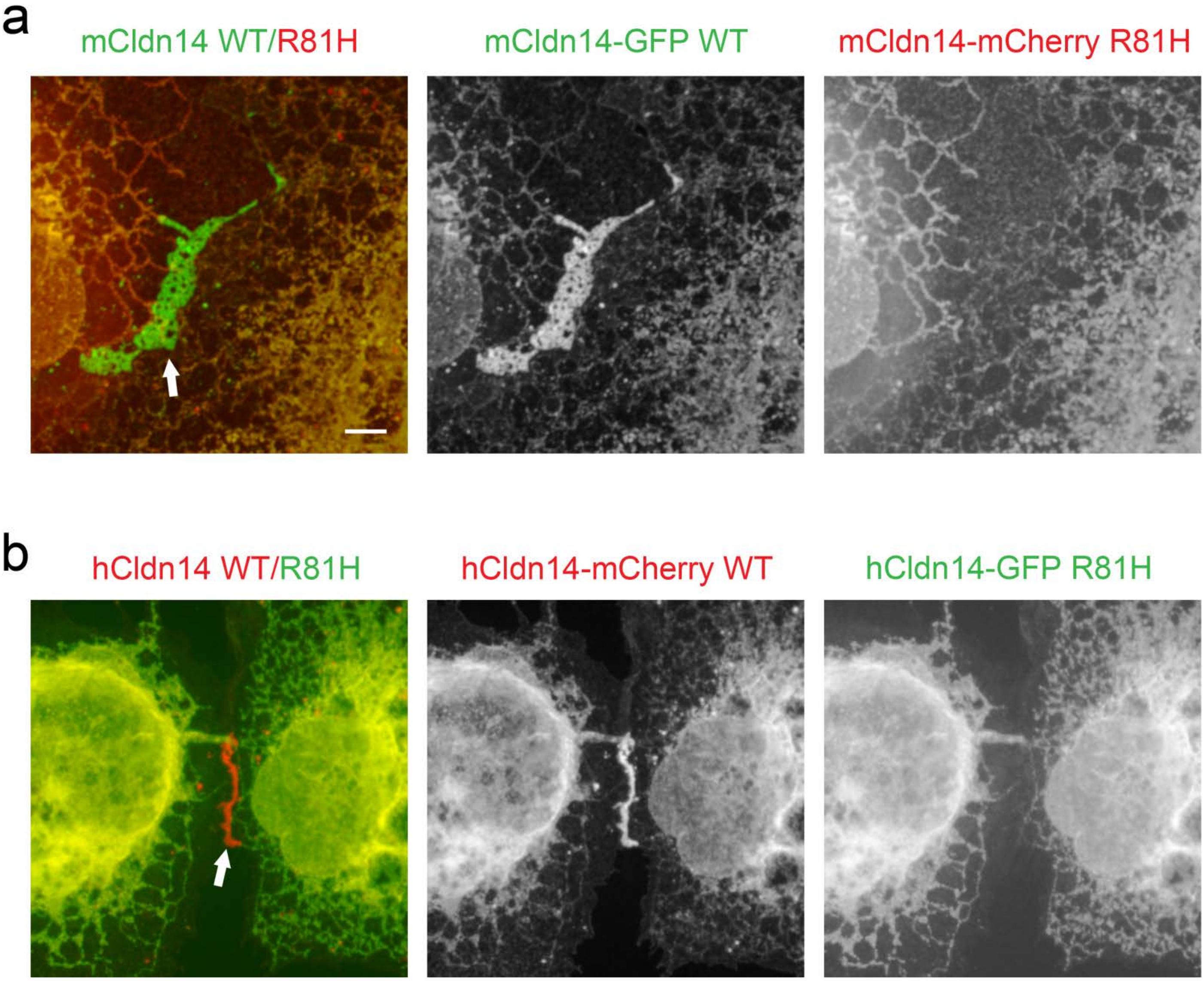

Supplementary Figure 11. Cldn14 R81H is excluded from TJs formed by Cldn14 WT. **a** Fluorescence confocal images of COS7 cells coexpressing mCldn14-GFP WT (green) and mCldn14-mCherry R81H (red). **b** Confocal imaging of COS7 co-expressing hCldn14-mCherry WT (red) and hCldn14-GFP R81H (green). Arrows indicate the position of TJs between transfected cells. Cldn14 WT and R81H mutants show similar colocalization except at the TJ where only the WT Cldn14 is observed. Scale bar = 5 µm.
