# Supplementary material for: Multiple claudin–claudin cis interfaces are required for tight junction strand formation and inherent flexibility
Source: Commun Biol. 2018 May 17;1:50. doi: 10.1038/s42003-018-0051-5 (PMC6123731; doi:10.1038/s42003-018-0051-5)
Supplement: Supplementary file 2 — Description of Additional Supplementary Files [file 42003_2018_51_MOESM2_ESM.docx]

**Description of Additional Supplementary Files**

File Name: Supplementary Movie 1

Description: Fluorescence confocal live-imaging of GFP-tagged Cldn2 strands. Confocal time series of individual TJ strands in Rat1 cells expressing mCldn2-GFP. The time stamp is indicated in the upper left. Scale bar = 2 µm.
